# Iron-dependent reconfiguration of the proteome underlies the intracellular lifestyle of *Brucella abortus*

**DOI:** 10.1038/s41598-017-11283-0

**Published:** 2017-09-06

**Authors:** M. S. Roset, T. G. Alefantis, V. G. DelVecchio, G. Briones

**Affiliations:** 1 Instituto de Investigaciones Biotecnológicas, Universidad Nacional de General San Martín, IIB-INTECH-CONICET, San Martín 1650, Buenos Aires, Argentina; 2 Vital Probes Inc., 1820 N. E.27th Drive, Wilton Manors, Florida USA; 3 Present Address: Sanofi Pasteur, 1 Discovery Drive, Swiftwater, PA USA

## Abstract

*Brucella* ssp. is a facultative intracellular pathogen that causes brucellosis, a worldwide zoonosis that affects a wide range of mammals including humans. A critical step for the establishment of a successful *Brucella* infection is its ability to survive within macrophages. To further understand the mechanisms that *Brucella* utilizes to adapt to an intracellular lifestyle, a differential proteomic study was performed for the identification of intracellular modulated proteins. Our results demonstrated that at 48 hours post-infection *Brucella* adjusts its metabolism in order to survive intracellularly by modulating central carbon metabolism. Remarkably, low iron concentration is likely the dominant trigger for reprogramming the protein expression profile. Up-regulation of proteins dedicated to reduce the concentration of reactive oxygen species, protein chaperones that prevent misfolding of proteins, and proteases that degrade toxic protein aggregates, suggest that *Brucella* protects itself from damage likely due to oxidative burst. This proteomic analysis of *B*. *abortus* provides novel insights into the mechanisms utilized by *Brucella* to establish an intracellular persistent infection and will aid in the development of new control strategies and novel targets for antimicrobial therapy.

## Introduction

*Brucella* is an intracellular bacterial pathogen that infects ruminants and other mammals as primary host producing abortions and reduction of fertility^1^. *Brucella* can also be transmitted to humans as a secondary host producing a worldwide disease named brucellosis, which is characterized by undulant fever and general malaise. Among all *Brucella* species, *B*. *abortus*, *B*. *melitensis* and *B*. *suis* have the most zoonotic prevalence^2^. The ability to survive within macrophages, a phagocytic host cell type which is the first line of defense against microbes, is critical for the persistence of *Brucella* within the host^3^. Due to its longstanding association with mammalian cells, *Brucella* has evolved molecular mechanisms to hijack host cell machinery and subvert programmed cell death, thereby allowing *Brucella* to survive within host cells^4, 5^. After macrophage internalization *Brucella* becomes enclosed in membrane bound compartment named as *Brucella*-containing vacuole (BCV). Initially, BCV interacts with early endosome vacuoles and later avoids the fusion to the highly degradative lysosome in order to survive. After 10–12 hours post-infection, those BCVs that have successfully escaped from entering the endocytic pathway fuse with endoplasmic reticulum-derived membranes becoming a permissive compartment for *Brucella* replication. At 24–48 hours post-infection, *Brucella* replicates actively within the cistern of an endoplasmic reticulum-like network that completely occupies the host cell cytosol^6^.

It has been reported that the activity of the *Brucella* type four protein secretion system (T4SS)^7^ and the production of periplasmic cyclic β1–2 glucans (CβG)^8, 9^ are important traits that allow the bacterium to successfully acquire its intracellular replicative niche within macrophages. T4SS is a “nanomachine” induced within the host cell^10^ that allows *Brucella* to translocate a battery of effector proteins to the host cell cytosol in order to modulate its normal physiology^11–16^. CβGs are critical for allowing BCVs to escape from the endocytic pathway, likely due to its ability to occlude cholesterol within its inner ring extracting it from membranes disrupting lipid rafts^17^. CβGs also have been involved in modulation of host inflammatory and immune responses^18, 19^.

To gain insights into the molecular mechanisms involved in intracellular adaptation and virulence of *Brucella*, a comparative proteomic investigation of *Brucella* grown in culture media or recovered from macrophages at 48 hours post-infection was performed using iTRAQ isobaric tags. This approach allowed a relative and absolute quantitation of modulated proteins from *in vitro-* and intracellular *Brucella* cells.

Our results indicate that *Brucella* undergoes an extensive rearrangement of its proteome in order to adapt to the intracellular microenvironment within macrophages.

## Results and Discussion

### The highly sensitive iTRAQ LC–MS/MS method allows the identification of *Brucella* proteins differentially expressed within macrophages

In this study, a global proteome analysis was conducted to directly investigate the differential proteomic profiles of *B*. *abortus* isolated within the host macrophage cell in comparison with *in vitro*-cultured *Brucella*. Here, the *Brucella* proteome was systematically analyzed using iTRAQ combined with liquid chromatography and MALDI TOF-TOF tandem mass spectrometry. This approach allows for the direct determination of relative protein abundance levels between comparative conditions and offers several advantages over traditional proteomic methodologies^20, 21^.

We selected a post-infection time of 48 hours when *Brucella* has already reached its replication niche in the ER-like compartment that occupied the whole host cell cytosol. This late post-infection time is particularly interesting because it allows us to understand the molecular mechanism that *Brucella* deploys in order to acquire its intracellular replicative niche within mammalian macrophage, a cell type which is responsible for engulfing and destroying bacterial pathogens.

**Figure 1:**
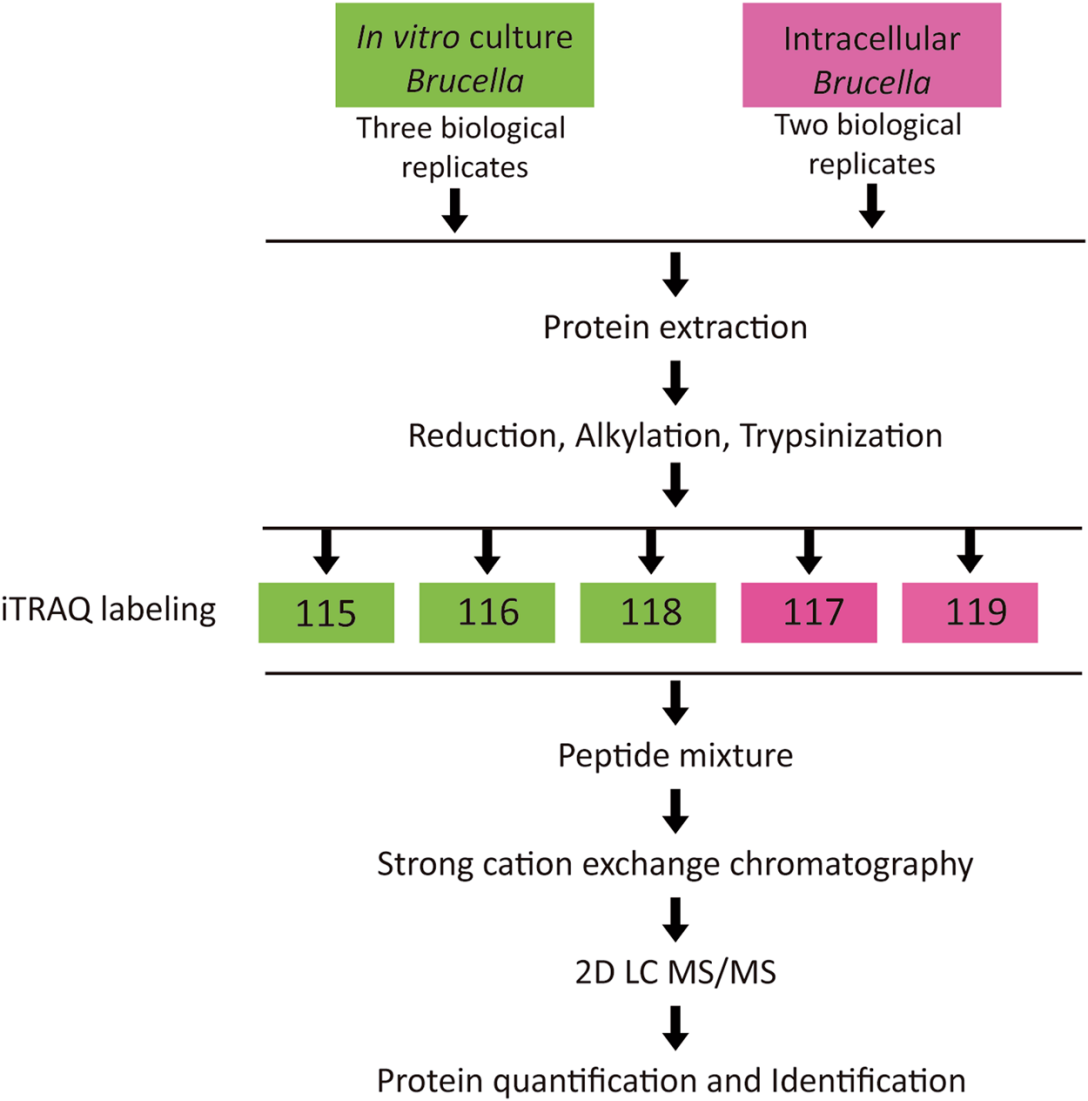
Schematic representation of the workflow of the iTRAQ experiment.

**Figure 2:**
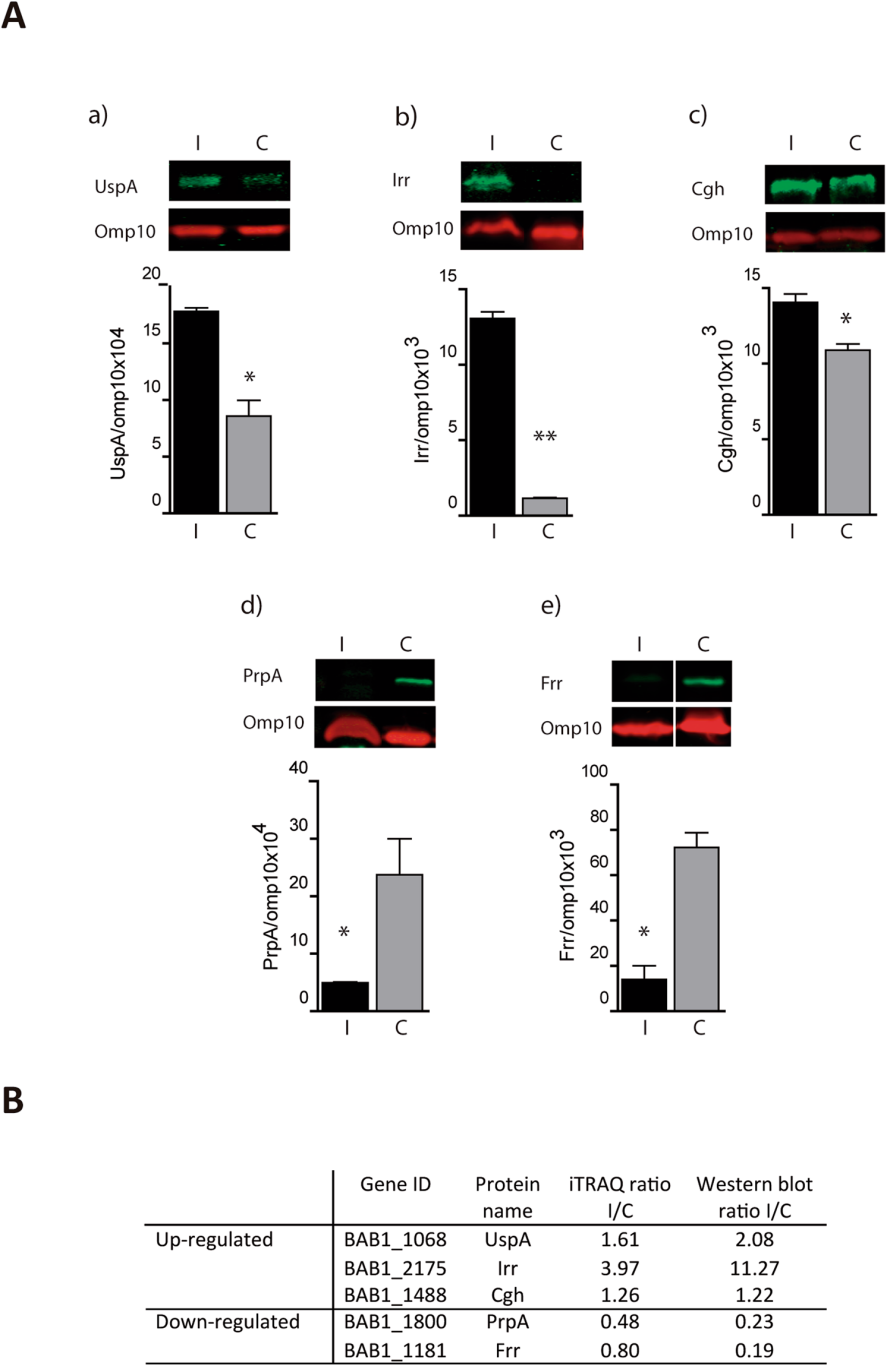
Western blot validation of up and down-regulated protein identified by iTRAQ proteomic. (**A**) Quantification of the intensity of bands (below) of the Western blots analysis (top) of the whole cell lysates of intracellular *Brucella* (I) or *Brucella* growth in culture media (control) (C). Blots were probed with mouse sera specific against; (a) universal stress protein (UspA), (b) ferric uptake regulator (Irr), (c) choloylglycine hydrolase (Cgh) (d) proline racemase (PrpA) and (e) ribosome recycling factor (Frr). Monoclonal antibody against anti-Omp10 was used as a normalizing control. Blots are representative of two independent experiments. Displayed Western blots bands were cropped from the original blot for clarity. Corresponding full-length blots are shown in Supplementary Figure S3. Unpaired two-tailed Student’s *t*-test (*P* < 0.05) was performed. (**B**) The table shows the comparison of the results of the mean ratio (I/C) obtained for iTRAQ or Western blot analysis.

**Figure 3:**
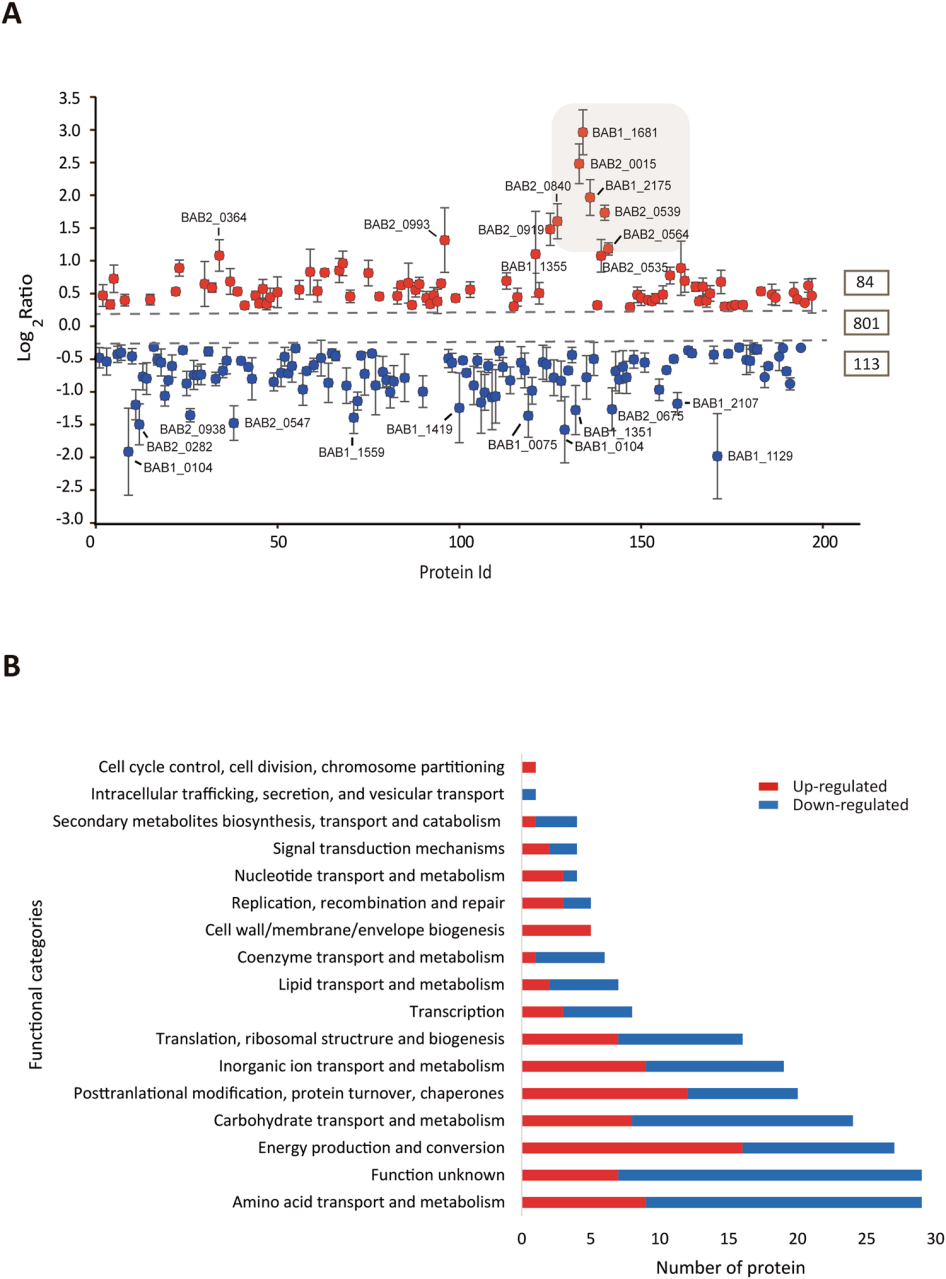
Global analysis of differentially expressed proteins. (**A**) Mean of log_2_ I/C ratio distribution of intracellular modulated proteins. The red and blue dots represent up-regulated and down-regulated proteins, respectively. Shade gray area represent proteins involved in iron transport and metabolism. Dash lines indicate the upper and lower log2 ratio cutoff (0.24, −0.26) used to consider proteins as being modulated. Numbers located at the right, indicate the total number of up, down and non-modulated proteins identified in this study. (**B**) Distribution of the identified modulated proteins accordingly to their assigned functional category (COGs).

A total of five independent iTRAQ experiments, two with intracellular *Brucella* (iTRAQ labels 117&119) and three with *in vitro*-cultured *Brucella* (iTRAQ labels 115, 116 & 118) were performed (Fig. 1), and a total number of 998 *Brucella* proteins were identified. Initially, from the five independent experiments, six ratio intracellular/*in vitro*-cultured control (I/C) were generated. To be considered as a differential expressed protein we selected the following criteria, (*i*) proteins with (I/C) ratios above 1.1 and below 0.9, (*ii*) protein ratios (I/C) with *P* values < 0.05, (*iii)* differentially expressed in at least two independent ratios (I/C), *iv*) proteins that displayed discrepancy in ratio direction (up or down regulated) were not considered. From this initial analysis, 207 proteins were identified as intracellular modulated proteins. To add a biological dimension to the initial cutoff value, proteins with I/C ratios values closer to the initial cutoff were selected and a Western blot analysis was performed to confirm their status as intracellular modulated protein. Using antibodies against ribosome recycling protein (Frr) with I/C ratios of 0.83 and 0.75 (Fig. 2A and Supplementary Table S1) or against choloylglycine hydrolase (Cgh) with I/C ratios of 1.18, 1.27 and 1.33 (Fig. 2A and Supplementary Table S1), a new cutoff value was selected in order to include as modulated proteins all the I/C ratios present in Frr and Cgh (I/C ratio below 0.83 or above 1.18). Considering this new cutoff, in addition of the rest of criteria mentioned before, 197 proteins were characterized as intracellular modulated protein, with 84 proteins being up-regulated and 113 down-regulated (Fig. 3A and Supplementary Table S1). Representative MS/MS spectra of peptides from differentially expressed proteins along with the intensity of their reporter ions (iTRAQ labels) are shown in Supplementary Fig. S1. Modulated proteins were assigned to functional categories using the cluster of orthologous groups (COGs) analysis. As shown in Fig. 3B, most of the proteins were grouped into those involved in nutrient transport (amino acids, carbohydrates & inorganic ions), metabolism (carbohydrates, amino acids), protein homeostasis (proteases, chaperones, ribosomal proteins, etc.), and ATP production (Tables 1, 2, 3, 4 and 5 and S2, S3 and S4).

**Table 1:** Inorganic ion transport and metabolism (P)^a^.

NCBI GI	Gene ID	Gene name	Protein description	Loc^b^	Signal peptide	Mean ratio(I/C)	SD	Ratio
**Iron transport and metabolism**								
GI:82616559	BAB1_1681	tonB	Periplasmic protein TonB	U	NO	7.94	1.74	↑
GI:82616997	BAB1_2150	dps	DNA starvation/stationary phase protection protein	C	NO	0.59	0.14	↓
GI:82617022	BAB1_2175	Irr	Ferric-uptake regulator	U	NO	3.96	0.75	↑
GI:82939245	BAB2_0015	entC	Isochorismate synthase	C	NO	5.68	1.18	↑
GI:83269064	BAB2_0113		ABC-type Fe3+ transport system, periplasmic component	U	YES	1.25	0.04	↑
GI:82939710	BAB2_0539	fbpA	ABC-type Fe3+ transport system, periplasmic component	P	YES	3.34	0.27	↑
GI:82939733	BAB2_0564	fatB	ABC-type enterochelin transport system, periplasmic component	P	YES	2.27	0.15	↑
GI:83269559	BAB2_0675	bfr	Bacterioferritin	C	NO	0.43	0.10	↓
GI:82939977	BAB2_0840		Protein probably involved in high-affinity Fe2 + transport	U	YES	3.08	0.59	↑
**Superoxide dismutase, Fe-Mn family**								
GI:82699463	BAB1_0591	sodA	Manganese and iron superoxide dismutase	P	NO	0.63	0.02	↓
**Superoxide dismutase, Cu-Zn family**								
GI:83269434	BAB2_0535	sodC	Copper/Zinc superoxide dismutase	P	NO	2.12	0.36	↑
**Lipoprotein**								
GI:82700922	BAB1_2176		Lipoprotein YaeC family:NLPA lipoprotein	CM	YES	0.71	0.12	↓
GI:83269197	BAB2_0275		YaeC family lipoprotein	CM	YES	0.59	0.11	↓
**Sulfate transport system**								
GI: 82699026	BAB1_0104		Sulfate-/thiosulfate-binding protein	P	YES	0.35	0.13	↓
GI: 82700154	BAB1_1351		Sulfate-/thiosulfate-binding protein	P	YES	0.42	0.10	↓
**Sulfonate/nitrate transport system**								
GI:82699122	BAB1_0214		putative hydroxymethylpyrimidine transport system substrate-binding protein	U	YES	0.57	0.08	↓
GI:83269772	BAB2_0919		Sulfonate/nitrate ABC transporter periplasmic-binding protein	U	YES	2.82	0.46	↑
GI:83269977	BAB2_1146		Solute-binding family 1 protein	U	YES	0.57	0.12	↓
**Other**								
GI:82616005	BAB1_1075		rhodanese family protein	P	NO	0.74	0.06	↓

^a^Abbreviations of assigned functional categories (http://www.ncbi.nih.gov/COG/).

^b^Abbreviations of cellular location. Protein cellular location was annotated by PSORTB v. 3.0 (http://www.psort.org/). C, cytoplasmic; P, periplasmic; U, unknown; CM, cytoplasmic

membrane.

Mean ratio (I/C), indicates the mean values of all I/C ratios with *P* < 0.05.

SD, indicates standard deviation of the mean.

**Table 2:** Energy production and conversion (C)^a^.

NCBI GI	Gene ID	Gene	Protein description	Loc.^b^	Signal peptide	Mean ratio(I/C)	SD	Ratio
**NADH dehydrogenase complex (Complex I)**								
GI:82615783	BAB1_0825	nuoD	NADH dehydrogenase subunit D	C	NO	0.62	0.10	↓
GI:82699683	BAB1_0826	nuoE	NADH dehydrogenase subunit E	C	NO	0.57	0.04	↓
GI:82615785	BAB1_0827	nuoF	NADH dehydrogenase subunit F	C	NO	0.50	0.08	↓
GI:82699685	BAB1_0828	nuoG	NADH dehydrogenase subunit G	C	NO	0.56	0.09	↓
**Cytochrome b-c1 complex (Complex III)**								
GI:82616452	BAB1_1559	petA	Rieske iron-sulfur domain	CM	NO	0.39	0.06	↓
**Cytochrome c oxidase (Complex IV)**								
GI:82615378	BAB1_0389	ccoP	cytochrome c heme-binding domain	CM	NO	0.61	0.11	↓
**F0F1 ATP Synthase (Complex V)**								
GI: 82700580	BAB1_1808		F0F1 ATP synthase subunit gamma	U	NO	1.55	0.01	↑
GI: 82700581	BAB1_1809	atpA	F0F1 ATP synthase subunit alpha	C	NO	1.39	0.12	↑
GI: 82700582	BAB1_1810	atpH	F0F1 ATP synthase subunit delta	C	NO	0.59	0.15	↓
**Tricarboxylic acid (TCA) cycle**								
GI: 82699012	BAB1_0090	AcnA	aconitate hydratase	C	NO	0.54	0.11	↓
GI: 82616088	BAB1_1170	gltA	citrate synthase	C	NO	1.38	0.12	↑
GI: 82700033	BAB1_1221		isocitrate dehydrogenase	C	NO	1.31	0.13	↑
GI: 82616792	BAB1_1926	sucC	Succinyl-CoA synthetase subunit beta	C	NO	1.58	0.02	↑
**Pyruvate dehydrogenase complex**								
GI: 82699966	BAB1_1149	E3	dihydrolipoamide dehydrogenase E3	C	NO	1.82	0.23	↑
GI: 82699967	BAB1_1150	E2	branched-chain alpha-keto acid dehydrogenaseE2	C	NO	1.37	0.09	↑
GI: 82699968	BAB1_1151	E1	pyruvate dehydrogenase subunit beta E1	C	NO	1.96	0.26	↑
GI: 82699969	BAB1_1152	E1	dehydrogenase, E1 component	C	NO	1.77	0.23	↑
**Oxoglutarate dehydrogenase complex**								
GI: 82700688	BAB1_1922	sucB	dihydrolipoamide acetyltransferase	C	NO	1.61	0.35	↑
GI: 82700689	BAB1_1923	sucA	alpha-ketoglutarate decarboxylase	C	NO	1.25	0.02	↑
**Others**								
GI: 82698965	BAB1_0036		cytochrome c heme-binding site:cytochrome c, class IA/ IB	CM	NO	0.51	0.08	↓
GI:82615422	BAB1_0435		FAD linked oxidase C	C	NO	1.37	0.03	↑
GI:82700730	BAB1_1971	etfA	electron transfer flavoprotein alpha subunit	U	NO	1.47	0.11	↑
GI:82700731	BAB1_1972	etfB	electron transfer flavoprotein subunit beta	C	NO	1.56	0.11	↑
GI: 83269185	BAB2_0261	pntA	RecA DNA recombination protein:alanine dehydrogenase/PNT	CM	NO	1.26	0.01	↑
GI: 83269262	BAB2_0346		3-isopropylmalate dehydrogenase	C	NO	1.46	0.17	↑
GI: 83269417	BAB2_0518		bifunctional proline dehydrogenase	C	NO	0.73	0.01	↓
GI: 83269759	BAB2_0904	narG	nitrate reductase alpha subunit	U	NO	0.62	0.13	↓

^a^Abbreviations of assigned functional categories (http://www.ncbi.nih.gov/COG/).

^b^Abbreviations of cellular location. Protein cellular location was annotated by PSORTB v. 3.0 (http://www.psort.org/). C, cytoplasmic; P, periplasmic; U, unknown; CM, cytoplasmic

membrane.

Mean ratio (I/C), indicates the mean values of all I/C ratios with *P* < 0.05.

SD, indicates standard deviation of the mean.

**Table 3:** Carbohydrate transport and metabolism (G)^a^.

NCBI GI	Gene ID	Gene	Protein description	Loc.^b^	Signal peptide	Mean ratio(I/C)	SD	Ratio
**Glycolysis/Gluconeogenesis**								
GI:82699048	BAB1_0128		zinc-containing alcohol dehydrogenase	C	NO	1.35	0.10	↑
GI:82699119	BAB1_0211		aldehyde dehydrogenase	U	NO	0.75	0.02	↓
GI:91206675	BAB1_0316	pgi	Glucose-6-phosphate isomerase	C	NO	0.60	0.06	↓
GI:82699972	BAB1_1155	eno	enolase	U	NO	1.60	0.40	↑
GI:82698932	BAB1_1742	pgk	Phosphoglycerate kinase	C	NO	1.45	0.02	↑
GI:82700535	BAB1_1761	pyk	pyruvate kinase	U	NO	1.51	0.07	↑
GI:83269246	BAB2_0327		aldehyde dehydrogenase	U	NO	0.46	0.04	↓
GI:83269280	BAB2_0364		fructose-1,6-bisphosphatase	U	NO	2.13	0.35	↑
GI:83269281	BAB2_0365	fbaA	fructose-1,6-bisphosphate aldolase	U	NO	1.86	0.16	↑
**Pentose Phosphate Shunt**								
GI:82700516	BAB1_1740	tkt	transketolase	C	NO	1.25	0.04	↑
GI:83269362	BAB2_0459	pgl	6-phosphogluconolactonase	U	NO	0.63	0.05	↓
GI:83269363	BAB2_0460		glucose-6-phosphate 1-dehydrogenase	U	NO	0.70	0.09	↓
**ATP-binding cassette ABC transporter complex**								
GI:82699142	BAB1_0238		ABC-type sugar transport system, periplasmic comp.	U	YES	0.60	0.09	↓
GI:82699145	BAB1_0241		ABC-type sugar transport systems, ATPase comp.	U	NO	0.60	0.06	↓
GI:82700425	BAB1_1648		ABC-type sugar transport system, periplasmic comp	U	NO	0.55	0.07	↓
GI:83269394	BAB2_0491		ABC-type sugar transport system, periplasmic comp.	U	NO	1.62	0.21	↑
GI:82939718	BAB2_0547		Probable sugar-binding periplasmic protein precursor	U	YES	0.36	0.07	↓
GI:83269478	BAB2_0585	ugpB	transport system substrate-binding protein	P	YES	0.65	0.01	↓
GI:83269790	BAB2_0938		ABC-type xylose transport system, periplasmic comp.	N	YES	0.39	0.03	↓
**Acting on carbohydrates and derivatives**								
GI:82700159	BAB1_1356	fucU	RbsD or FucU transport	C	NO	0.78	0.01	↓
**Inositol phosphate metabolism**								
GI:82700499	BAB1_1723		inositol phosphatase/fructose-1,6-bisphosphatase	C	NO	0.77	0.04	↓
**Galactose metabolism**								
GI:83269216	BAB2_0294		dihydroxy-acid dehydratase	U	NO	0.62	0.01	↓
GI:83269217	BAB2_0295		2-keto-3-deoxy-galactonokinase	U	NO	0.57	0.04	↓
**Propanoate metabolism**								
GI:83269853	BAB2_1009	mgsA	methylglyoxal synthase	C	NO	0.69	0.01	↓

^a^Abbreviations of assigned functional categories (http://www.ncbi.nih.gov/COG/).

^b^Abbreviations of cellular location. Protein cellular location was annotated by PSORTB v. 3.0 (http://www.psort.org/). C, cytoplasmic; P, periplasmic; U, unknown; CM, cytoplasmic

membrane.

Mean ratio (I/C), indicates the mean values of all I/C ratios with *P* < 0.05.

SD, indicates standard deviation of the mean.

**Table 4:** Translation, ribosomal structure and biogenesis (J)^a^.

NCBI GI	Gene ID	Gene	Protein description	Loc.^b^	Signal peptide	Mean ratio(I/C)	SD	Ratio
**Ribosomal proteins**								
GI:82615467	BAB1_0480	rpsF	30S ribosomal protein S6	C	NO	0.80	0.01	↓
GI:82700001	BAB1_1184	rpsB	30S ribosomal protein S2	C	NO	0.73	0.10	**↓**
GI:82700063	BAB1_1251	rpsS	30S ribosomal protein S19	C	NO	0.79	0.02	↓
GI:82616165	BAB1_1252	rplB	50S ribosomal protein L2	C	NO	0.62	0.02	↓
GI:82616167	BAB1_1254	rplD	50S ribosomal protein L4	C	NO	0.54	0.03	↓
GI:91207342	BAB1_1267	rplA	50S ribosomal protein L1	C	NO	0.78	0.04	↓
GI:82700871	BAB1_2124	rplT	50S ribosomal protein L20	C	NO	0.59	0.07	↓
**Others**								
GI:82699075	BAB1_0159	raiA	protein/ribosomal protein S30EA	U	NO	0.66	0.02	↓
GI:82699088	BAB1_0172	Rph	ribonuclease PH	C	NO	1.54	0.11	↑
GI:82699769	BAB1_0918	gatB	aspartyl/glutamyl-tRNA amidotransferase subunit B	C	NO	1.41	0.15	↑
GI:82699781	BAB1_0930		ribonuclease E and G	C	NO	1.36	0.11	↑
GI:82699998	BAB1_1181	frr	ribosome recycling factor	C	NO	0.79	0.04	↓
GI:82700035	BAB1_1223	alaS	alanyl-tRNA synthetase	C	NO	1.33	0.04	↑
GI:82700083	BAB1_1271	ef_Tu	elongation factor Tu	U	NO	1.45	0.04	↑
GI:82616743	BAB1_1872	prfA	peptide chain release factor 1	C	NO	1.43	0.16	↑
GI:82939410	BAB2_0198		16S rRNA uridine-516 pseudouridylate synthase	C	NO	1.28	0.02	↑

^a^Abbreviations of assigned functional categories (http://www.ncbi.nih.gov/COG/).

^b^Abbreviations of cellular location. Protein cellular location was annotated by PSORTB v. 3.0 (http://www.psort.org/). C, cytoplasmic; P, periplasmic; U, unknown; CM, cytoplasmic

membrane.

Mean ratio (I/C), indicates the mean values of all I/C ratios with *P* < 0.05.

SD, indicates standard deviation of the mean.

**Table 5:** Posttranslational modification, protein turnover, chaperones (O)^a^.

NCBI GI	Gene ID	Gene	Protein description	Loc.^(b)^	Signal peptide	Mean ratio (I/C)	SD	Ratio
**Oxidoreductases-Peroxidases**								
GI:82699792	BAB1_0941	bcp	Alkyl hydroperoxide reductase	C	NO	0.75	0.03	↓
GI:83269430	BAB2_0531	ahpC	Alkyl hydroperoxide reductase	C	NO	1.92	0.59	↑
GI:82939705	BAB2_0532	ahpD	alkylhydroperoxidase, AhpD	U	NO	1.48	0.12	↑
GI:83269711	BAB2_0848		Catalase	P	YES	0.63	0.14	↓
**Chaperones and folding catalysts**								
GI:82699086	BAB1_0170	grpE	Molecular chaperone GrpE	C	NO	1.40	0.13	↑
GI:82699227	BAB1_0333	Hsp33	Hsp33-like chaperonin	C	NO	1.62	0.20	↑
GI:82699332	BAB1_0446	dnaJ	heat shock protein DnaJ, N-terminal	C	NO	0.78	0.01	↓
GI:82699709	BAB1_0855	grxD	glutaredoxin:glutaredoxin-related protein	U	NO	0.63	0.01	↓
GI:82700211	BAB1_1413	degP	Serine protease family protein	P	YES	1.30	0.02	↑
GI:82700479	BAB1_1703	ftsH	peptidase M41	C	YES	1.34	0.07	↑
GI:82699956	BAB1_1138	clpA	chaperonin ClpA/B	C	NO	1.23	0.05	↑
GI:82700707	BAB1_1944	ppiC	PpiC-type peptidyl-prolyl cis-trans isomerase	CM	YES	1.30	0.04	↑
GI:82700858	BAB1_2107	Trx-1	thioredoxin domain-containing protein	C	NO	0.44	0.05	↓
GI:82700877	BAB1_2130	dnaJ	cytochrome c heme-binding domain-containing protein	C	NO	0.51	0.06	↓
**Iron-sulfur cluster assembly**								
GI:82699057	BAB1_0139	nifU	nitrogen-fixing NifU, C-terminal	C	NO	1.52	0.07	↑
GI:82699799	BAB1_0948	sufB	cysteine desulfurase activator complex subunit	U	NO	1.72	0.15	↑
**Peptidases**								
GI:82699040	BAB1_0118		Zn peptidase superfamily	C	NO	1.39	0.16	↑
GI:82699578	BAB1_0710		leucyl aminopeptidase	P	NO	0.75	0.06	↓
GI:82700211	BAB1_1413	degP	Serine protease family protein	P	YES	1.30	0.02	↑
GI:82700479	BAB1_1703	ftsH	peptidase M41	C	YES	1.34	0.07	↑
GI:82616716	BAB1_1845	ctpA	carboxyl-terminal protease	CM	NO	1.36	0.13	↑
GI:82939548	BAB2_0358	dcp	Peptidyl dipeptidase DCP	C	NO	0.59	0.08	↓

^a^Abbreviations of assigned functional categories (http://www.ncbi.nih.gov/COG/).

^b^Abbreviations of cellular location. Protein cellular location was annotated by PSORTB v. 3.0 (http://www.psort.org/). C, cytoplasmic; P, periplasmic; U, unknown;

CM, cytoplasmic membrane.

Mean ratio (I/C), indicates the mean values of all I/C ratios with *P* < 0.05.

SD, indicates standard deviation of the mean.

### The expression profile of proteins modulated within macrophages suggests that BCV is an iron-limiting compartment

As shown in Table 1, the comparison of the protein expression profile of *Brucella* residing within the host cell versus proteins expressed by the bacteria grown in culture medium showed a differential expression of several proteins involved in iron transport and metabolism. Remarkably, the master regulator Irr (Iron-responsive regulator protein) (BAB1_2175), which is responsible for sensing variations in iron concentration, was highly up-regulated in the BCV. *Brucella* Irr has been reported to be stable in iron-limiting growth conditions and degraded when iron is available^22^. As a consequence of its stabilization, Irr promotes the induction of iron acquisition systems. Accordingly, isochorismate synthase (BAB2_0015), one of the enzymes required for *Brucella* siderophore biosynthesis, was detected up-regulated (Table 1). Siderophores are small iron-chelators molecules that the bacteria excrete to the extracellular medium to capture iron from the environment. The induction of the operon responsible for siderophore synthesis was also observed in an intracellular *Brucella* transcriptome analysis^23^. Iron-loaded siderophores are transported back to the bacteria mediated by specific ABC transporters. The uptake of ferri-siderophores by the bacteria is challenging since it requires passage through the bacterial outer and inner membranes, a process that proceeds against an electrochemical gradient in the absence of any ATP production^24^. Thus, bacteria have evolved proteins like the periplasmic TonB that energize the process of transporting across the membrane by transducing a proton motive force generated in the inner membrane. Similar to the previously reported transcriptomic study^23^, TonB (BAB1_1681) was the protein most over-expressed in intracellular *Brucella* (Table 1 and Fig. 3A, see shaded area). No changes were observed in the expression of its inner membrane partners ExbB (BABI_1679) and ExbD (BABI_1680). Ferric uptake through the outer membrane can be mediated by siderophores or by hijacking host ferric-binding proteins such as transferrin or lactoferrin mediated by receptors. Once the iron cargo is incorporated either by siderophores or by siderophore-independent pathways, a periplasmic transferrin-like protein named FbpA, captures free iron for further delivery to the cytosol mediated by ferric-specific ABC transporters^25^. We observed that *Brucella* FbpA (BAB2_0539) was up-regulated within the host cell. In addition, we also detected the intracellular up-regulation of an enterochelin-like ABC transporter (BAB2_0564), a periplasmic component of a Fe^3+^ transport system (BAB2_0113), and a Fe^2+^ specific ABC transporter (BAB2_0840). Moreover, *Brucella* iron storage proteins, bacterioferritin (BAB2_0675) and Dps (BAB1_2150) were down-regulated. Interestingly, in *Bradyrhizobium*, the gene transcription of bacterioferritin is repressed when the bacterium grows in iron restricted condition in an Irr-dependent manner^26^. A recent report showed that *Salmonella* downregulated Dps in response to oxidative stress^27^. The iron related proteins described above had the highest level of up-regulation in this proteomic analysis (Fig. 3A, see shaded area). All these results showed that *Brucella* is subjected to iron deprivation within the BCV microenvironment.

At this point it was interesting to evaluate the modulation of *Brucella* iron-regulated proteins also at transcriptional level. A set of these proteins were selected to study their modulation by using RT-qPCR. As shown in Supplementary Fig. S2 *B*. *abortus* recovered from macrophages induced the transcription of *irr*, *entC* genes, and reduced the expression of *dps* gene. Therefore, modulation in the expression level of Irr, EntC and Dps proteins can be explain as a consequence of the induction or repression of their respective genes.In a previous report Martinez *et al*.^22^ also observed the up-modulation of Irr when *B*. *abortus* was grown in an iron-depleted culture medium. They further concluded that up-regulation of Irr was not due to the induction of *irr* transcription (using a transcriptional fusion approach) but rather a consequence of a reduction of Irr degradation. Here, in addition of detecting the up-modulation of Irr protein by iTRAQ, we also observed the induction of *irr* gene transcription in intracellular *Brucella* by RT-qPCR as shown above. Apparent discrepancies between both observations at transcriptional level it might be partially explained by differences in sensitivity of the used techniques in both cases. However, it would be interesting to speculate that transcriptional and translational up-regulation that was detected here it might be the result of a long-term iron deprivation found by *Brucella* when located within the host cells for 48 hours, compared with the *in-vitro* iron deprivation condition. Interestingly, in an intracellular *Brucella melitensis* and *Brucella canis* transcriptomic report^23^, it was found that the transcription of *irr* gene was reduced at 24 hours post-infection. If differences observed by Eskra *et al*.^23^ compared with our results are due to a differential regulation of *irr* transcription in different species of *Brucella* or it is simply a consequence of an alternative experimental setting (24 hour post-infection and RAW cells) it remains to be explored further.

### Iron scarcity promotes a reduction in the activity of the TCA cycle and oxidative phosphorylation

**Figure 4:**
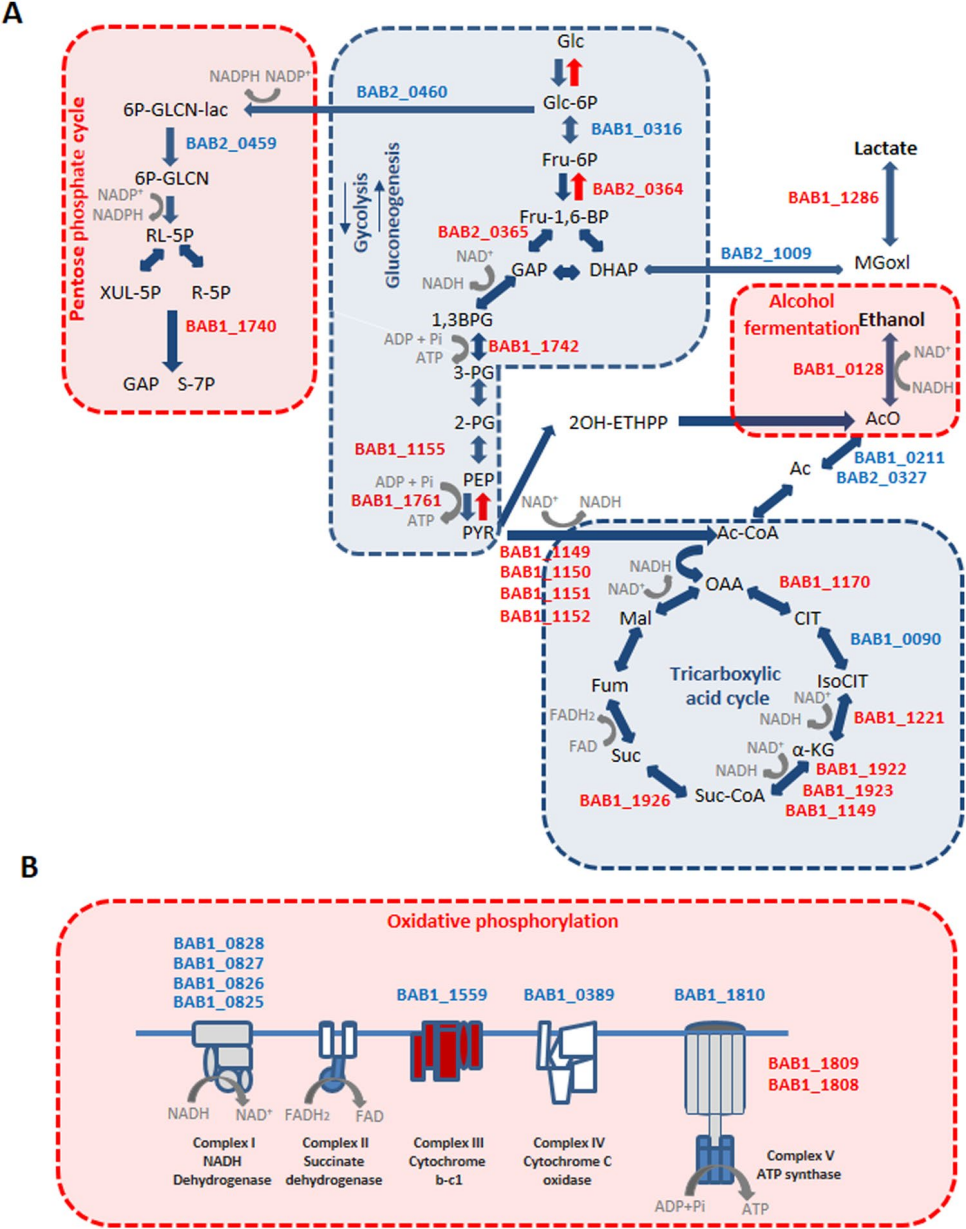
Protein expression changes of metabolic pathways involved in (**A**) central carbon metabolism or (**B**) oxidative phosphorylation in *B*. *abortus* during its intracellular life. Locus Tags depicted either in red or blue indicate up or down-regulated proteins respectively.

It has been reported that iron deprivation can lead to a perturbation of energy producing metabolic pathways. This is due to the fact that enzymes of these pathways contain iron-sulfur (Fe-S) proteins or heme-containing enzymes which require iron for their activity^28^. Accordingly, we found in this study, that *Brucella* aconitase (BAB1_0090), one of the critical enzymes from the TCA cycle, was severely down-regulated within the host cell (Table 2 and Fig. 4). Similar findings were reported in the bacterium *Staphylococcus* or in the fungus *Paracoccidioides brasiliensis* when iron source is limited^29, 30^. Bacterial aconitases are iron-sulfur proteins that have been described as moonlighting or multitasking proteins that in addition to their metabolic role in TCA and glyoxylate cycles, also have a role as mRNA-binding proteins that modulates post-transcriptionally the expression of proteins involved in iron metabolism^31^.

ATP and NADH are produced during glycolysis and in the TCA cycle. In addition to its role in redox reaction enzymes (especially in catabolism), NADH is also the first electron donor of the oxidative phosphorylation chain, at Complex I (NADH:ubiquinone oxidoreductase) or Nuo complex. The Nuo complex plays a central role in the generation of ATP, since it couples electron transference between NADH and ubiquinone to proton translocation^32, 33^. We found that all the subunits of the dehydrogenase domain (NuoE, NuoF and NuoG) (BAB1_0826, BAB1_0827, and BAB1_0828, respectively) and one subunit of the connecting domain (NuoD, BAB1_0825) of Nuo Complex I were down-regulated (Table 2 and Fig. 4). Interestingly NuoE, NuoF and NuoG possess iron sulfur clusters, while the connecting domain protein NuoD has a Ni-Fe cluster. All these results also suggest that down-regulation of Complex I proteins is related to iron deficiency.

In addition to the down-regulation of the Nuo complex, components of Complex III (PetA, BAB1_1559) and Complex IV (CcoP, BAB1_0389), an iron sulfur protein and a heme-containing protein, respectively, were also down regulated. It has been reported in bacteria and fungus that the oxidative phosphorylation chain is interrupted when microorganisms are grown in an iron-free medium, with a consequent reduction in ATP production^29, 30^. The last component of the oxidative phosphorylation chain is the F_0_F_1_ complex that utilizes the proton gradient associated to the electron transfer to phosphorylate ADP. As protons in the periplasm are transported back to the cytosol, conformational changes in F_0_F_1_ ATP synthase promote ATP production. When oxidative phosphorylation is interrupted, F_0_F_1_ still has the ability to reverse the reaction of H^+^ extrusion from cytosol by consuming ATP, a process which is critical for adaptation of bacteria to acidic environment^34^. As shown in Table 2, we detected that the alpha (BAB1_1809) and gamma (BAB1_1808) subunits of the F_0_F_1_ complex were up-regulated in intracellular *Brucella*. It is conceivable to speculate that up-regulation of F_0_F_1_ might reflect a requirement of ATP hydrolysis to generate a proton gradient across the inner membrane and to allow *Brucella* to maintain a physiological intracellular pH.

It was reported that microorganisms reinforce the use of metabolic pathways such as glycolysis when they face limitations in energy production (e.g., as a consequence of reduction in the oxidative phosphorylation) since they can synthesize ATP by substrate-level phosphorylation^29, 30^. We have also identified an up-regulation of four enzymes of glycolysis in intracellular *Brucella* (Fig. 4 and Table 3): fructose-1,6-bisphosphate aldolase (BAB2_0365), phosphoglycerate kinase (BAB1_1742), enolase (BAB1_1155), and pyruvate kinase (BAB1_1761). Both phosphoglycerate kinase and pyruvate kinase contribute to ATP formation by substrate-level phosphorylation, thereby preventing an energy shortage if the synthesis of ATP through oxidative phosphorylation is impaired.

As mentioned above, ATP and NADH are the main products of glycolysis. Since aerobic glucose oxidation is not proceeding at the level of the respiratory chain, ATP demand lead to an increased metabolism due to incremented glycolysis and consequently an accumulation of NADH. Along with NADH accumulation, NAD^+^ is not regenerated which is a critical cofactor required for an effective continuation of glycolysis. Interestingly, BAB1_0128, a zinc-containing alcohol dehydrogenase, an enzyme that consumes NADH to produce ethanol and regenerate NAD^+^ was detected up-regulated in intracellular *Brucella* (Table 3 and Fig. 4). The increased activity of this alcohol dehydrogenase could contribute to maintain the redox balance by recycling NADH. Methylglyoxal is another product derived from glycolysis which is produced from dihydroxyacetone phosphate by the action of methylglyoxal synthase. Accumulation of metylglyoxal is very toxic for the cell and there are glyoxalase enzymes (glyoxalases 1 and 2) that metabolize this compound to lactate^35^. In intracellular *Brucella*, methylglyoxal synthase (BAB2_1009) (Table 3 and Fig. 4) is down-regulated while glyoxalase (BAB1_1286) (Supplementary Table S2 and Fig. 4) is up-regulated, suggesting that intracellular *Brucella* is controlling the increase in concentration of this compound to avoid toxicity.

Pyruvate dehydrogenase complex (PDH) plays a key role in carbon metabolism. It transform pyruvate derived from glycolysis into acetyl-coenzyme A that can either enter the TCA cycle or be available to enter a number of alternative pathways such as fatty acids or amino acids biosynthesis. As shown in Table 2 and Fig. 4, all the subunits of PDH (BAB1_1149, BAB_1150, BAB1_1151 and BAB1_1152) were up-regulated by *Brucella* within the host cell. This has also been reported in the closely-related α2-proteobacteria *Sinorhyzobium meliloti* in which the PDH complex is also up-regulated when the bacterium is located intracellularly within the plant cell^36^.

### Living within the macrophage is a stressful condition for *Brucella abortus*

At 48 hours post-infection, a global down-regulation of the expression of *Brucella* ribosomal protein (r-proteins) was observed suggesting a reduction of protein synthesis (Table 4). Reduction of the r-proteins content has been linked to the stringent response (SR), the adaptive response to different types of starvation stresses^37^. A recent transcriptomic study of intracellular *Brucella* also found a critical down-regulation in r-protein transcription^38^. A set of *Brucella* proteins characterized as stress adaptation proteins were also modulated within the macrophage. This included the universal stress protein UspA (BAB1_1068) (Supplementary Table S4) which was reported to respond to different types of stressors (temperature, oxidative and osmotic) in the human pathogen *Escherichia coli* O157:H7^39^.

Another critical challenge that intracellular bacteria have to face is the oxidative stress response within professional macrophages. As shown in Table 5, both components of the *Brucella* alkyl hydroperoxide reductase complex AhpC (BAB2_0531) and AhpD (BAB2_0532), which are involved in detoxification of H_2_O_2_ produced by oxidative burst in macrophages were up-regulated. In addition, an enzyme that quenches superoxide anion radicals (O_2_^−^) such as superoxide dismutase C (BAB2_0535) was also up-regulated (Table 1). In contrast, the Fe-S containing enzyme catalase (BAB2_0848) (Table 5) was down-regulated, probably also as a consequence of iron deprivation as discussed above. Oxidative stress promotes bacterial protein denaturation and the formation of protein aggregates which are toxic for bacterium. To restore protein homeostasis, a set of bacterial proteins that assist the process of protein refolding (chaperones) or degradation of protein aggregates (proteases) were over-expressed. As shown in Table 5, three major proteases HtrA (BAB1_1413), CtpA (BAB1_1845) and FtsH (BAB1_1703) and a putative protease M20/M25/M40 (BAB1_0118) were all up-regulated in intracellular *Brucella*. Depending on their activity, molecular chaperones have been classified in i) “folding chaperones” (*e*.*g*., DnaK and GroEL), ii) “holding chaperones” (*e*.*g*., Hsp33, Hsp31 and IbpB) that maintain unfolded proteins in solution awaiting availability of “folding” chaperones, and iii) chaperones that solubilize protein aggregates produced by stress (*e*.*g*., ClpB). Although DnaK, a critical bacterial chaperone, was not modulated, its cochaperones GrpE (BAB1_0170) and DnaJ (BAB1_0446) were up-regulated and down-regulated, respectively (Table 5). Interaction of DnaK chaperone with its cochaperones GrpE and DnaJ determines the avidity of DnaK for misfolded proteins. When the nucleotide exchange factor GrpE replace ADP by ATP on DnaK, this chaperone is locked in an “open” conformation, with low affinity for peptides. This conformation favors the release of peptides allowing DnaK to start a new cycle of folding. It has been described that over-expression of GrpE tends to inhibit DnaK function^40, 41^. Whether the combination of GrpE up-regulation and DnaJ down-regulation reflects a reduced DnaK activity remains to be determined.

Another chaperone, Hsp33 (BAB1_0333), was found to be up-regulated. It has been described that Hsp33 is inactive when its four cysteins located in the active site are in a reduced state. As a consequence of oxidative stress, the cysteins become oxidized leading to the activation of Hsp33 that form dimers or oligomers that bind to protein targets^42^. As shown in Table 5, the chaperone ClpA (BAB1_1138) was also up-regulated. This protein is critical in *Brucella suis* to survive against oxidative stress^43^. We also observed the up-regulation of a peptidyl-prolyl-cis-trans isomerase, PpiC (BAB1_1944), which is involved in protein folding. In a recent report we showed that cyclophilins CypA and CypB (one type of peptidyl-prolyl-cis-trans isomerase enzymes) were also up-regulated intracellularly playing a critical role in adaptation of *Brucella* within host cells^44^. The up-regulation of proteins dedicated to reduce the concentration of reactive oxygen species, protein chaperones that prevent misfolding of proteins, and proteases that degrade toxic protein aggregates, suggest that *Brucella* is adapting to a stressful condition within macrophages, protecting itself from damage likely due to oxidative burst.

### Validation of proteomic results

To confirm iTRAQ results by an alternative method such as Western blot analysis we selected five proteins that were modulated intracellularly (three up and two down-regulated). As a reference internal control (RIC), the protein Omp10 was used because (i) it was not modulated in our proteomic study, (ii) it was not reported to be affected in different *Brucella* proteomic studies and (iii) it is an immunogenic protein that allow us to use it as a reference with high sensitivity. The amount of modulated protein in comparison with RIC in both intracellular and *in vitro* conditions is shown in Fig. 2A. The results were in accordance with those observed in the iTRAQ analysis (Fig. 2B) showing that intracellular *Brucella* (relative to the *in vitro* control) contains increased amounts of universal stress protein (UspA, BAB1_1068), ferric-uptake regulator (Irr, BAB1_2175) or choloylglycine hydrolase (Cgh, BAB1_1488) and decreased amounts of prolil racemase (PrpA, BAB1_1800) or ribosome recycling factor (Frr, BAB1_1181).

In addition to the required validation of proteomic results from a statistical point of view or by Western blot analysis, it is interesting to analyze our results regarding metabolic or functional criteria. For instance, here we observed that all the subunits of different protein complexes (or most of them) show a coordinated modulation. Thus, the PDH complex (Table 2), the F_0_F_1_ ATP synthase complex (Table 2), the 2-oxoglutarate dehydrogenase complex (Table 2) and the electron transfer flavoprotein complex (Table 2) displayed a coordinated up-regulation. In addition, several ribosomal proteins (Table 4) and the Nuo complex (Table 2) were down-regulated. An interesting observation was that proteins of two different systems dedicated to the same function, the Fe-S cluster assembly, SufB (Table 5) and Nif U (BAB1_ Table 5), were both up-regulated.

### Iron concentration is an important signal that *Brucella* sense for intracellular adaptation

As shown in Supplementary Table S5, a more detailed analysis determines that about the 28% of modulated proteins were somehow related to iron metabolism or regulated by iron concentration. In addition, our results highlighted the critical role of the master regulator Irr also in intracellular adaptation of *Brucella*. Accordingly, Irr was up-regulated at transcriptional and translational level in intracellular *Brucella* (Table 1 and Figs 2 and S2). It has been described that Irr is able to recognize and bind a DNA sequence motif named ICE-Box (also known as Irr binding motif)^26, 45, 46^. Irr can act either as an activator or repressor factor depending on the proximity of ICE consensus motif to the promoter of the Irr-regulated genes^26^. A search for ICE consensus motif was performed along the intergenic regions of intracellularly modulated proteins using the Find Individual Motif Ocurrences (FIMO) algorithm software (Supplementary Table S5)^47^. Of interest, from the 197 modulated proteins, 55 of them (28%) were proteins related to iron, from which 35 have also a predicted ICE motif, a strong suggestion that Irr is playing a central role in intracellular adaptation (Supplementary Table S5).

### Proteomic studies showed common features in microbial pathogen-host interactions

There are common features in the intracellular protein expression profile of different intracellular pathogens such as *Shigella*, *Salmonella* or *Brucella*. Intracellular *Salmonella* showed an up-regulation of proteins involved in iron transport and metabolism^48^ while intracellular *Shigella* modifies its central carbon metabolism with reduction of oxidative phosphorylation^49^, as described here for *Brucella*.

**Figure 5:**
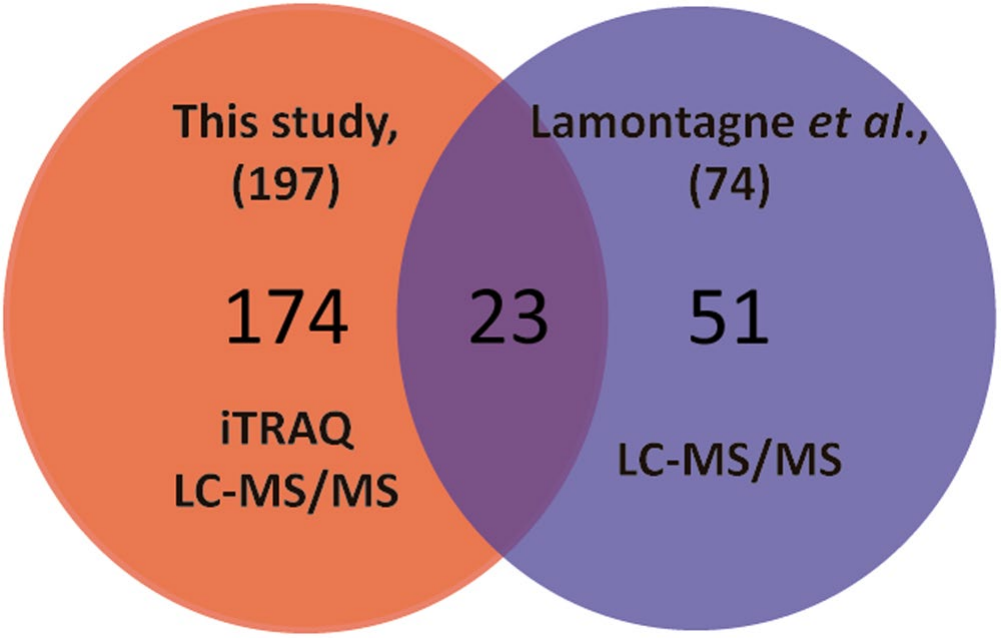
Comparison of the results of previous intracellular proteomic report in *B*. *abortus* 2308 with our study. Intersection of the Venn diagram shows common differentially expressed proteins identified in both studies.

Results depicted in Fig. 5, showed the comparison of our results with a previous intracellular proteomic report in *Brucella abortus* 2308 at 44 hours post-infection^50^. Intersection in the Venn diagram representation shows common proteins that were differentially expressed intracellularly (23 proteins) in both studies (Fig. 5). Only one protein showed discrepancy in the direction of the modulation (up or down regulation) between both reports.

In this report we have identified a set of 174 new differentially modulated proteins in intracellular *Brucella*. We believe these results are providing new insights into the molecular mechanisms that the bacterium utilizes in order to adapt to its intracellular replication niche within macrophages.

## Concluding Remarks

Of the total number of proteins that *B*. *abortus* modulates within macrophages, 28% of them were either related with iron transport, iron storage, iron as cofactor, the iron master regulator Irr and proteins which expression is controlled by Irr. As reported in an earlier publication^51^ *Brucella* grown in iron-restricted conditions had a greater invasiveness and virulence. This observation is particularly interesting because allow us to speculate if iron scarcity in addition of being a nutritional stressor it might also function as a “positioning” signal that tells *Brucella* about its intracellular localization within host cells. Results presented here are consistent with the idea that iron deprivation is an important cue that conduit *B*. *abortus* to shape its proteome in order to increase its intracellular fitness. Here, by using the highly sensitive iTRAQ/MS/MS technique, we provided new insights into the *Brucella* virulence mechanisms that will redounds in the design of new strategies to control this worldwide zoonosis.

## Materials and Methods

### Growth of *B*. *abortus*

All experiments involving live *Brucella abortus* 2308 (*Brucella melitensis biovar* Abortus 2308) were conducted under Biosafety level 3 (BSL3) conditions. *B*. *abortus* strains were grown in *Brucella* broth (BB) (Difco Laboratories, Detroit, Mich.) at 37 °C on a rotary shaker (250 rpm). Culture media were supplemented with nalidixic acid (Nal) at 5 μg/ml.

### Cell culture and infection assay

Cell culture and infection assay of J774 macrophages for proteomic studies was performed as described previously^44^. Aliquots of the resulting lysates were serially diluted in PBS and plated on BB agar to determine CFUs.

### Isolation of *Brucella* from infected J774 macrophages

Isolation of *Brucella* from infected macrophages was performed as described previously^44^. Infected cell lysates were centrifuged at 210 × g for 10 min at 4 °C to remove host cell debris. The culture supernatant was collected and centrifuged at 20,000 × g for 30 min at 4 °C, and the resulting pellet was resuspended in 3 ml of Tris-sucrose (TS) buffer (33 mM Tris-hydrochloride containing 0.25 M sucrose [pH 7.4]). Three milliliters of the bacterial suspension were loaded onto 27 ml of Percoll (GE Healthcare Life Sciences) prepared at 30% (vol/vol) in polycarbonate centrifuge tubes. Tubes were centrifuged at 25,000 × g for 60 min at 4 °C to allow the development of a self-forming-gradient by isopycnic centrifugation leading to the development of two gradient bands. The lower band of the gradient, containing more than 85% of *Brucella* cells, was collected and diluted 10-fold in ice-cold PBS (pH 7.4) and then centrifuged at 20,000 × g for 30 min at 4 °C. The pellet was resuspended in PBS and recentrifuged to eliminate residual Percoll. The final pellet from each gradient was resuspended in PBS, and protein content and *Brucella* viability were determined. In addition, bacteria *in vitro* cultured were subjected to the same purification steps.

### Protein extraction

Protein extraction was performed as described previously^44^. Protein extraction from *Brucella* isolated from macrophages or from *in vitro*-cultured bacteria was performed in the same manner. Aliquots of 75 ul of *Brucella* cells suspension were mixed with 37.5 ul of acetonitrile (ACN) (to a final concentration of 7.5% (vol/vol)) and 387.5 ul of 8 M urea in a total volume of 500 μl. The cells were sonicated using 5 pulses of 5-s duration each with a 30-s rest between each pulse. After sonication, the cells were centrifuged (15,000 x g, 10 min, 4 °C) and the supernatant was removed and kept. The supernatant containing 0.8 mg/ml of protein was then applied to a Pall 10 K Nanosep column and concentrated to approximately 75 μl. A series of buffer exchange and protein cleaning steps were performed as follows, with reconcentration to 75 μl after each step: step 1, addition of 4% CHAPS {3-[(3-cholamidopropyl)-dimethylammonio]-1-propanesulfonate} buffer (500 μl); step 2, addition of 7.5% ACN plus 4% CHAPS (500 μl); step 3, addition of 4% CHAPS (500 μl) followed by a repeat of step 3; step 4, addition of 0.05% CHAPS (500 μl) followed by a repeat of step 4. After each extraction, the total protein concentration was determined by Bradford analysis.

### Trypsin Digest and iTRAQ Labeling

For trypsin digest 100 µg of protein from either intracellular or *in vitro* grown *Brucella* were aliquoted and dried down in a centrivap. The protein pellet was then resuspended in 20 µl of Dissolution Buffer (0.5 M triethylammonium bicarbonate (TEAB) (diluted to 0.5 M with dH_2_O, pH 5.5). Denaturant (1 µl of 2% (wt/vol) SDS) was then added and the sample was vortexed. Then the reducing reagent tris-(2-carboxyethyl) phosphine (TCEP) (1 µl, final concentration 5 mM) was added, the sample was vortexed and centrifuged briefly, and then incubated at 60 °C for 1 hour. Then, fresh iodoacetamide (1 µl of an 84 mM stock) was added and the sample was wrapped in aluminum foil and incubated at room temperature for 30 minutes. Sequencing grade trypsin (Promega) was then reconstituted with Resuspension buffer (50 mM acetic acid), and 2 µl of the resulting suspension was added to the protein sample and the entire reaction was incubated overnight at 48 °C. Samples were then dried in a vacuum concentrator to a total volume of no more than 33 µl.

For this analysis, the 8-plex iTRAQ kit was utilized (Applied Biosystems). Trypsin digests from two intracellular and three *in vitro Brucella* samples were labeled with iTRAQ according to the manufacture’s protocol. Briefly, all iTRAQ reagents were brought to room temperature and then 50 µl of isopropanol was added to each. The entire contents of each resuspended iTRAQ label was then added to a sample vial and incubated at room temperature for 2 hours. Then, 100 µl of dH_2_O was added to quench each reaction and incubated for an additional 30 minutes at room temperature. All iTRAQ labeled samples (5 samples including labels 115, 116, 117, 118 and 119) were then combined into a single tube and dried down in a speed vac. Chasing of the sample was then performed by three rounds of resuspension in 100 µl dH_2_O and drying in a speed vac. The final dried sample was then submitted to The Pennsylvania State University Hershey Medical Center Mass Spectrometry Core Facility (Hershey, PA) for LC-MS/MS analysis.

### 2D-LC Separations

Strong cation exchange (SCX) separations were performed on a passivated Waters 600E HPLC system using a 4.6 × 250 mm PolySULFOETHYL Aspartmide column (PolyLC, Columbia, MD) at a flow rate of 1 ml/min. Buffer A contained 10 mM ammonium formate, pH 2.7, in 20% acetonitrile/80% water. Buffer B contained 666 mM ammonium formate, pH 2.7, in 20% acetonitrile/80% water. The gradient was Buffer A at 100% (0–22 minute following sample injection), 0–40% Buffer B (16–48 min), 40–100% Buffer B (48–49 min), then isocratic 100% Buffer B (49–59 min), then at 56 min switched back to 100% Buffer A to re-equilibrate for the next injection. The first 26 ml of eluant (containing all flow-through fractions) was combined into one fraction, then 14 additional 2-ml fractions were collected. All 15 of these SCX fractions were dried down completely to reduce volume and to remove the volatile ammonium formate salts, then resuspended in 9 μl of 2% (vol/vol) acetonitrile, 0.1% (vol/vol) trifluoroacetic acid and filtered prior to reverse phase C18 nanoflow-LC separation.

For the 2^nd^ dimension separation by reverse phase nanoflow LC, each SCX fraction was auto injected onto a Chromolith CapRpd column (150 × 0.1 mm, Merck) using a 5-μl injector loop on a Tempo LC MALDI Spotting system (ABI-MDS/Sciex). Buffer C was 2% acetonitrile, 0.1% trifluoroacetic acid, and Buffer D was 98% acetonitrile, 0.1% trifluoroacetic acid. The elution gradient was 95% C/5% D (2 μl per minute flowrate from 0–3 min, then 2.5 μl per minute from 3–8.1 mon), 5% D to 38% D (8.1–40 min), 38% D to 80% D (41–44 min), 80% D to 5% D (44–49 min). Flow rate was 2.5 μl/min during the gradient, and an equal flow of MALDI matrix solution was added post-column (7 mg/ml recrystallized CHCA (α-cyano-hydrocinnamic acid), 2 mg/ml ammonium phosphate, 0.1% trifluoroacetic acid, 80% acetonitrile). The combined eluant was automatically spotted onto a stainless steel MALDI target plate every 6 seconds (0.6 μl per spot), for a total of 370 spots per original SCX fraction.

### Mass Spectrometry Analysis and Protein Identification and Quantitation

After the sample spots were dried on the MALDI plate, 13 calibrant spots (ABI 4700 Mix) were added to each plate manually. MALDI target plates (15 per experiment) were analyzed in a data-dependent manner on an ABI 4800 MALDI TOF-TOF. As each plate was entered into the instrument, a plate calibration/MS Default calibration update was performed and then the MS/MS default calibration was updated. MS spectra were then acquired from each sample spot using the newly updated default calibration using 500 laser shots per spot with a laser intensity of 3200. A plate-wide interpretation was then automatically performed, choosing the highest peak of each observed m/z value for subsequent MS/MS analysis. Up to 2500 laser shots at a laser power of 4200 were accumulated for each MS/MS spectrum. When the MS and MS/MS spectra from all 15 plated in a sample set were acquired, protein identification and quantitation were performed using the Paragon algorithm as implemented in the Protein Pilot 3.0 software (version 2.01 prior to July 2009, from ABI/MDS-Sciex). The spectra were searched against the NCBInr database for *Brucella abortus* plus common contaminants as well as a reversed “decoy” version of itself.

For the ProteinPilot analysis, the preset Thourough (iTRAQ or Identification) search settings were used and identifications must have a ProteinPilot Unused Score greater than 1.3 (95% Confidence interval) in order to be accepted. In addition, the only protein IDs accepted were required to have a “Local False Discovery Rate” estimation of no higher than 5%, as calculated from the slope of the accumulated Decoy database hits by the PSPEP (Proteomics System Performance Evaluation Pipeline) program.

### Western blot analysis for iTRAQ validation

Whole-cell lysates of the intracellular *B*. *abortus* and the *in vitro* growth control were subjected to 12% SDS-PAGE and transferred onto nitrocellulose membranes using a semidry transfer procedure. Immunoblotting was performed using as primary antibodies anti-UspA (1:500) (generated in this study), anti-Irr (1:4000) (kindly provided by M. Almirón), anti-PrpA (1:500) (kindly provided by JM Spera), anti-Frr (1:1000) (kindly provided by J. Cassataro), anti-Cgh (1:100) (kindly provided by MV Delpino) and anti-Omp10 (1:2000) (kindly provided by Axel Cloeckaert), and IRDye secondary anti-mouse or anti-rabbit antibodies (LI-COR, Inc.). All antibodies were diluted in TBS, 1% nonfat milk, 0.1% Tween solution. Detection was performed using the Odyssey imaging system (LI-COR, Inc.).

### RNA isolation and RTqPCR

Total RNA was isolated from intracellular and *in vitro Brucella* with Trizol Reagent according to manufacturer’s instructions (Life Technologies) and RNA integrity was evaluated by 1% agarose gel electrophoresis. Samples were incubated with RQ1 DNAse (Promega). One microgram of RNA was reverse transcripted using a commercial kit (Superscript II RT, Invitrogen, CA, USA) with 200 ng of random primers. qRT-PCR reactions were carried out using PowerUp^Tm^ SYBR Green Master Mix (Applied Biosystems, Foster City, CA) on the Step one plus Real time PCR system (Applied Biosystems, Foster City, CA). LinRegPCR software was used to analyze raw data and determinate efficiency of PCR reactions^52^. The relative expression of genes was calculated using Relative expression software tool (REST©)^53^. Real time quantitative PCR was performed with primers described below. Irr mRNA abundance was measured with the oligonucleotides F 5′-CACTTGCGAGCCTGATTTTC-3′ and R 5′-AGACCACATTTTCCCCTTCG-3. EntC mRNA was measured with the oligonucleotides F 5′-AAGCTTGGTCTGTACTTCGG-3′ and R 5′-TCGAGCGTGGATTGTTTACC-3′. Dps mRNA was measured with the oligonucleotides F 5′-GTGAAAATATCTGCCGTGTCG-3′ and R 5′-GGTGAAGGAATCCAGACTGAAG-3′. The relative quantity of 16S rRNA using the oligonucleotides F 5′-GAGTATGGAAGAGGTGAGTGG-3′ and R 5′-CAGGCGGAATGTTTAATGCG-3′, was used as endogenous control to normalize all the values. Each assay was performed in biological triplicates.

## Electronic supplementary material

Supplementary information
