# Supplementary material for: Iron-dependent reconfiguration of the proteome underlies the intracellular lifestyle of Brucella abortus
Source: Sci Rep. 2017 Sep 6;7:10637. doi: 10.1038/s41598-017-11283-0 (PMC5587712; doi:10.1038/s41598-017-11283-0)
Supplement: Supplementary file 1 — Supplementary information [file 41598_2017_11283_MOESM1_ESM.pdf]

Supplementary Information

Iron-dependent reconfiguration of the proteome underlies the intracellular lifestyle of *Brucella abortus*

Roset MS[1*], Alefantis TG[2], DelVecchio VG[2], Briones G[1*]**

Supplementary Figure S1

A AGQHPYNTALFADYIAHHGVAK(FbpA-BAB2_0539)

i)

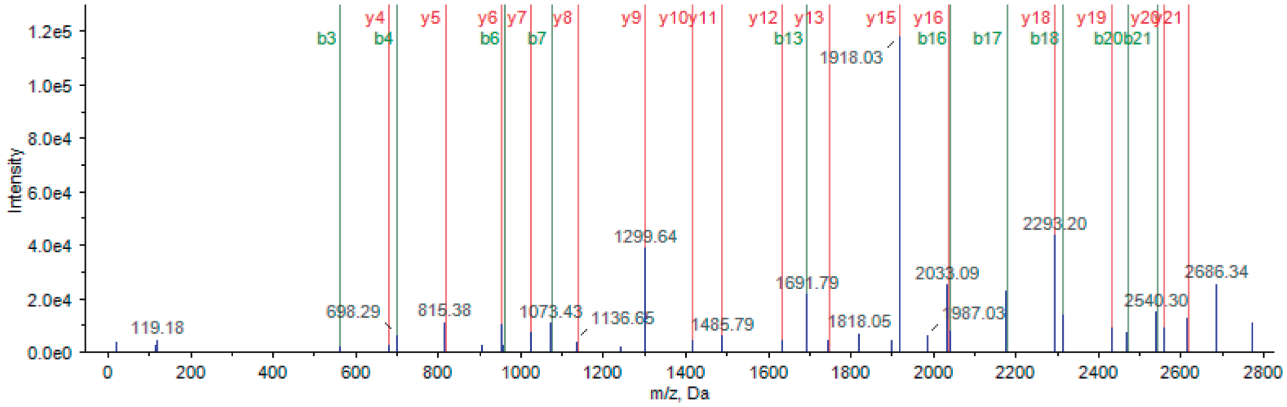

ii)

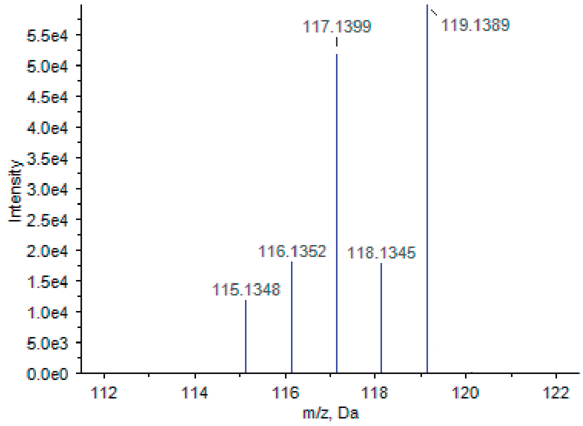

B EESIEEMHHADK(Bacterioferritin-BAB2_0675)

i)

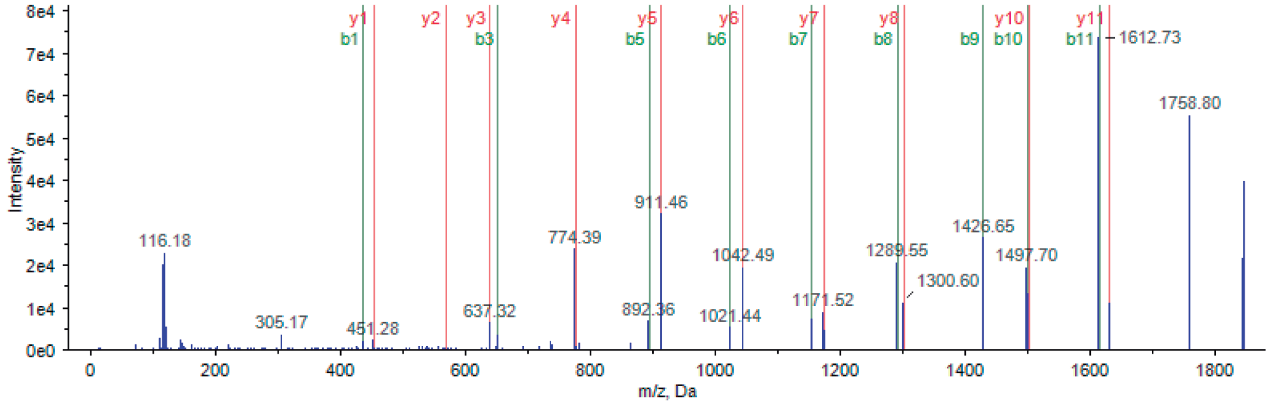

ii)

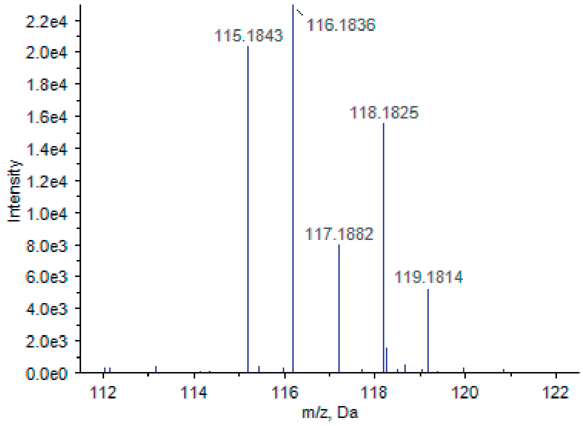

<u>Supplementary Figure S1:</u> Protein identification spectra of one selected peptide derived from the protein Solute binding protein family 1 (FbpA- BAB1_0539) (Panel A-i) or bacterioferritin (Bfr- BAB2_0675) (Panel B-i). Panel A-ii and B-ii showing the intensity of reporter ions and relative abundance of peptide-derived from FbpA and Bfr proteins indicating up- and down-regulation respectively.

Supplementary Figure S2

A

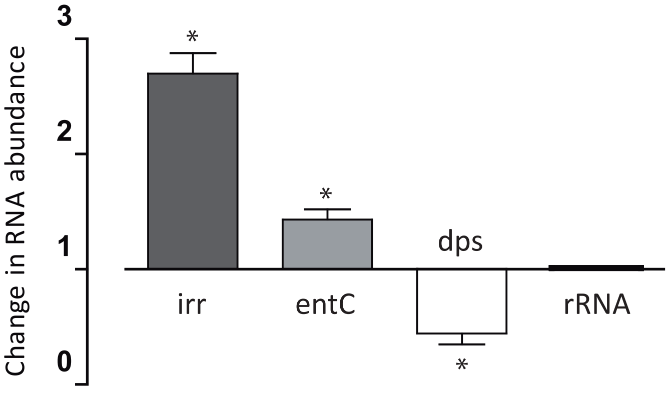

B

	Gene ID	Protein name	iTRAQ ratio I/C	QPCR ratio I/C
Up-regulated	BAB1_2175	Irr	3.97	2.70
	BAB2_0015	EntC	5.09	1.43
Down-regulated	BAB1_2150	Dps	0.59	0.44

Supplementary Figure S2: Real-time qPCR analysis of gene expression levels. *B. abortus* expression levels of *irr*, *entC* and *dps* genes in intracellular *Brucella* or *Brucella* growth in culture media (control) were determined and normalized to the expression of the 16S rRNA gene. (A) Values are expressed as mRNA relative abundance from intracellular/control assays. Data were obtained from at least three independent biological experiments. One sample t-test (P<0.05) was performed. (B) The table shows the comparison of the results of the mean ratio (I/C) obtained for iTRAQ or RTqPCR analysis.

Supplementary Figure S3

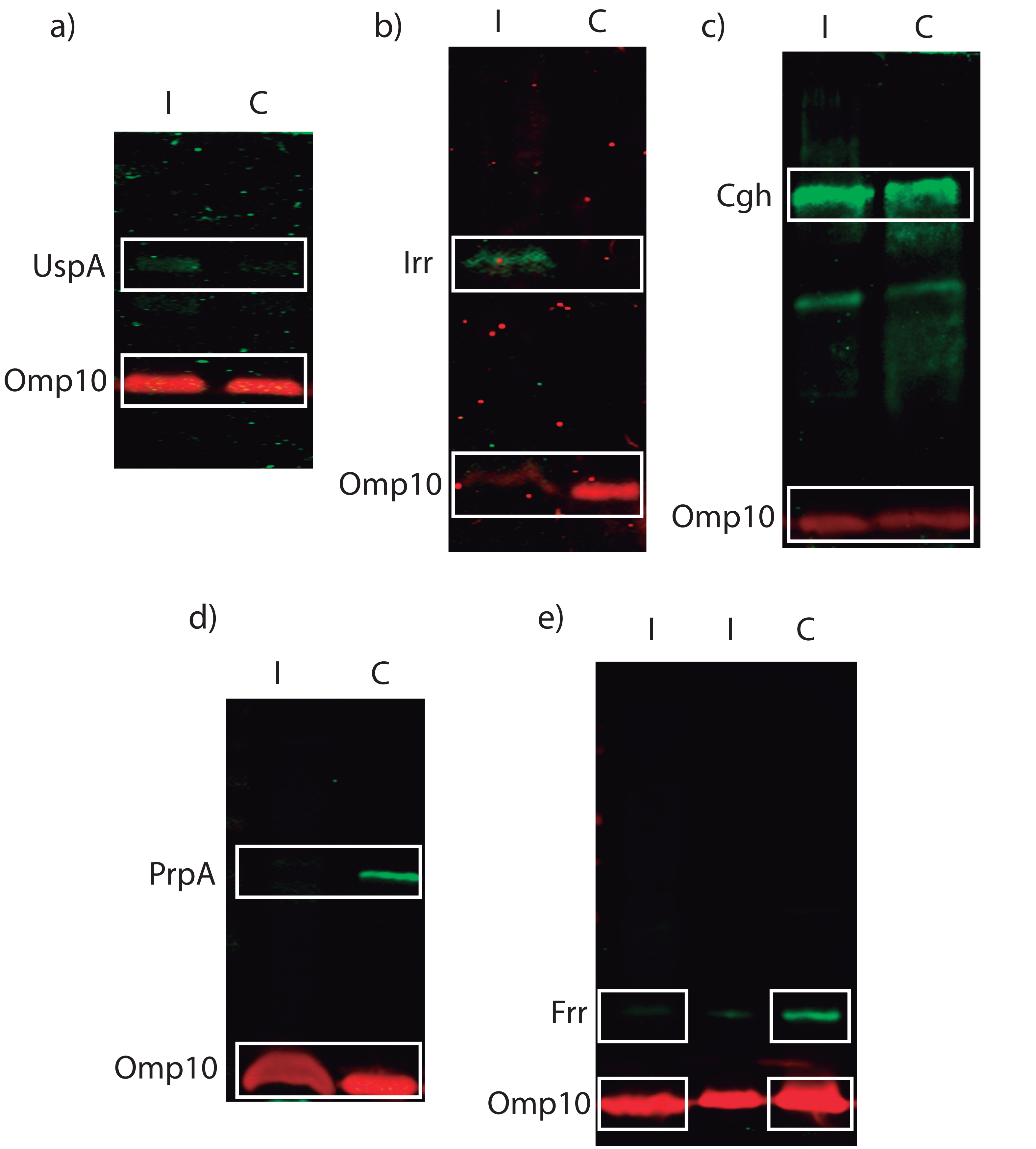

Figure S3: Western blot validation of up and down-regulated protein identified by iTRAQ proteomic. The figure shows the corresponding full-length blots of Figure 2. Western blots analysis of the whole cell lysates of intracellular *Brucella* (I) or *Brucella* growth in culture media (control) (C). Blots were probed with mouse sera specific against; (a) universal stress protein (UspA), (b) ferric uptake regulator (Irr), (c) choloylglycine hydrolase (Cgh) (d) proline racemase (PrpA) and (e) ribosome recycling factor (Frr). Monoclonal antibody against anti-Omp10 was used as a normalizing control.

Supplementary Table S1

Up-regulated and down-regulated proteins are highlighted in red and blue respectively.

Locus tag	Accession #	Protein description	117/115	P	119/115	P	117/116	P	119/116	P	117/118	P	119/118	P
BAB1_0002	gi|82698934	DNA polymerase III subunit beta					1.3788	0.0253			1.2495	0.0024		
BAB1_0005	gi|82615038	D-isomer specific 2-hydroxyacid dehydrogenase NAD-binding			0.8028	0.0207							0.7806	0.0386
BAB1_0036	gi|82698965	cytochrome c heme-binding site	0.5912	0.0344			0.4227	0.0114	0.5026	0.0519				
BAB1_0071	gi|82698997	argininosuccinate synthase	0.7798	0.0062			0.5693	0.001	0.6709	0.001	0.7548	0.001		
BAB1_0075	gi|82699001	hypothetical protein BAB1_0075					0.4562	0.042	0.3307	0.0225				
BAB1_0090	gi|82699012	aconitate hydratase 1	0.4647	0.0006	0.4969	0.001	0.653	0.0018	0.7017	0.0002	0.4538	0.0055	0.4831	0.001
BAB1_0096	gi|82699018	dihydroxy-acid dehydratase	0.4255	0.014	0.442	0.0227	0.6196	0.0227	0.6369	0.0355	0.4859	0.0164	0.5064	0.0191
BAB1_0104	gi|82699026	sulfate-/thiosulfate-binding protein	0.5129	0.0088	0.5368	0.0075	0.2665	0.0008	0.2587	0.0004	0.2752	0.0026	0.2687	0.0007
BAB1_0118	gi|82699040	hypothetical protein BAB1_0118	1.5193	0.0256	1.2187	0.0083	1.4376	0.0345						
BAB1_0128	gi|82699048	zinc-containing alcohol dehydrogenase							1.2811	0.0029			1.4159	0.0177
BAB1_0139	gi|82699057	nitrogen-fixing NifU, C-terminal					1.4682	0.0166	1.5707	0.0055				
BAB1_0159	gi|82699075	sigma 54 modulation protein/ribosomal protein S30EA									0.673	0.0174	0.6408	0.0232
BAB1_0170	gi|82699086	GrpE protein	1.2784	0.0004	1.2997	0.0002	1.5113	0.001	1.521	0.001				
BAB1_0172	gi|82699088	ribonuclease PH	1.4656	0.0261			1.6164	0.0349						
BAB1_0204	gi|82699116	zinc-containing alcohol dehydrogenase	0.667	0.0003			0.4287	0.0379	0.4636	0.0401			0.3012	0.0455
BAB1_0211	gi|82699119	aldehyde dehydrogenase							0.7615	0.0241	0.7285	0.0059	0.7585	0.0072
BAB1_0214	gi|82699122	sulfonate/nitrate transport system substrate-binding protein					0.5829	0.0434	0.4877	0.0108			0.6476	0.0345
BAB1_0215	gi|82699123	thiamine-phosphate pyrophosphorylase					0.5777	0.0175	0.4952	0.0132	0.618	0.0092	0.5362	0.0003
BAB1_0238	gi|82699142	solute-binding family 1 protein	0.6888	0.0496	0.6264	0.0015	0.5279	0.0021	0.4898	0.0001			0.684	0.003
BAB1_0241	gi|82699145	ABC transporter ATPase					0.5317	0.0069	0.5832	0.0291	0.6056	0.0246	0.6731	0.0146
BAB1_0285	gi|91207052	Histidinol dehydrogenase			1.3277	0.0212	1.1914	0.0417	1.2686	0.0124	1.212	0.007	1.309	0.0012
BAB1_0316	gi|91206675	Glucose-6-phosphate isomerase	0.678	0.028	0.6009	0.0216	0.5782	0.0072	0.5035	0.0062	0.6672	0.0176	0.5943	0.0194
BAB1_0333	gi|82699227	Hsp33-like chaperonin					1.7624	0.0195			1.4848	0.0422		
BAB1_0368	gi|82699260	invasion protein B			0.7012	0.0146	0.4267	0.0251	0.359	0.0047			0.4808	0.0102
BAB1_0389	gi|82699277	cytochrome c heme-binding site	0.6452	0.0462	0.748	0.0115					0.4889	0.0153	0.564	0.0012
BAB1_0435	gi|82699321	FAD linked oxidase	1.3512	0.0076							1.394	0.0006		
BAB1_0446	gi|82699332	heat shock protein DnaJ, N-terminal:chaperone DnaJ, C-terminal:heat shock protein DnaJ			0.7661	0.0229			0.7854	0.0481				
BAB1_0480	gi|82615467	30S ribosomal protein S6					0.7953	0.0258	0.7985	0.0033				
BAB1_0484	gi|82699370	acyl carrier protein	2.1083	0.0378			1.498	0.0495						
BAB1_0591	gi|82699463	manganese and iron superoxide dismutase					0.636	0.0014			0.6048	0.0321	0.6383	0.0004
BAB1_0636	gi|82699504	response regulator receiver:transcriptional regulatory protein, C terminal					0.6244	0.0333	0.8171	0.0372				
BAB1_0657	gi|82699525	histidinol-phosphate aminotransferase					1.5963	0.0422			1.9408	0.0459	1.4626	0.0179
BAB1_0660	gi|82699528	Omp2b porin	1.214	0.0255							1.5733	0.0465		
BAB1_0688	gi|82699556	dihydroorotase	1.2728	0.031	1.3377	0.0002					1.4549	0.0098	1.5314	0.0003
BAB1_0710	gi|82699578	leucyl aminopeptidase	0.7538	0.0118							0.6776	0.0231	0.8048	0.0025
BAB1_0776	gi|82699635	hypothetical protein BAB1_0776			1.4888	0.0004	1.2071	0.0466	1.4486	0.0021			1.321	0.003
BAB1_0780	gi|82699639	delta-aminolevulinic acid dehydratase							0.7778	0.0282			0.6757	0.0155
BAB1_0787	gi|82615747	Serine hydroxymethyltransferase					1.4528	0.0281	1.4358	0.0143				
BAB1_0788	gi|91208373	Transcriptional repressor nrdR	1.225	0.0341			1.2745	0.0267			1.2143	0.0114		
BAB1_0799	gi|82699658	integration host factor subunit alpha			1.3353	0.023							1.5011	0.0262
BAB1_0813	gi|82699672	O-acetylhomoserine aminocarboxypropyltransferase			0.7547	0.0153	0.6167	0.0293						
BAB1_0825	gi|82699682	NADH dehydrogenase subunit D	0.4835	0.0065	0.6081	0.0192	0.5576	0.0053	0.6943	0.0181	0.6176	0.002	0.7814	0.0162
BAB1_0826	gi|82699683	NADH dehydrogenase subunit E	0.5395	0.0497	0.5965	0.0072								
BAB1_0827	gi|82699684	NADH dehydrogenase I subunit F	0.4431	0.0182							0.561	0.0456		
BAB1_0828	gi|82699685	NADH dehydrogenase subunit G	0.4501	0.005	0.4561	0.0048	0.6516	0.0315	0.6553	0.0268	0.581	0.0119	0.5897	0.0083
BAB1_0855	gi|82699709	glutaredoxin:glutaredoxin-related protein									0.6272	0.0262	0.6332	0.0259
BAB1_0857	gi|82699711	phosphoribosylformylglycinamidine synthase II	1.4026	0.0081	1.2312	0.0182					1.4224	0.0222		
BAB1_0868	gi|82699722	adenylosuccinate lyase							1.2928	0.0302			1.3477	0.0034
BAB1_0873	gi|82699727	3-oxoacyl-(acyl carrier protein) synthase II					0.6142	0.0022	0.6756	0.0369	0.7053	0.0218		
BAB1_0918	gi|82699769	aspartyl/glutamyl-tRNA amidotransferase subunit B	1.2663	0.0099	1.2817	0.0017	1.5357	0.0005	1.5398	0.0008				
BAB1_0930	gi|82699781	ribonuclease E and G					1.3932	0.0023			1.4418	0.0023	1.234	0.0349
BAB1_0941	gi|82699792	Alkyl hydroperoxide reductase/ Thiol specific antioxidant/ Mal allergen							0.7306	0.0024			0.7741	0.0052
BAB1_0948	gi|82699799	cysteine desulfurase activator complex subunit SufB					1.9047	0.01	1.6527	0.01	1.7586	0.0219	1.5499	0.0021
BAB1_0993	gi|82699830	unkwown	3.671	0.01	1.9269	0.01					2.1813	0.01		

BAB1_1015	gi|82699847	TatD-related deoxyribonuclease	1.4081	0.018	1.4034	0.0005	1.4678	0.0094	1.4457	0.0016	1.7101	0.0014	1.7106	0.0037
BAB1_1029	gi|82699861	hypothetical protein BAB1_1029	0.7603	0.034							0.6644	0.0151		
BAB1_1068	gi|82699897	universal stress protein	1.6996	0.0098	1.7374	0.0232					1.3878	0.0291		
BAB1_1073	gi|82699902	cysteine synthase A			0.8016	0.0229							0.7148	0.0082
BAB1_1075	gi|82616005	rhodanese family protein							0.8072	0.0066	0.7228	0.0231	0.6888	0.0088
BAB1_1109	gi|82699928	acyl-CoA dehydrogenase	0.583	0.0212	0.4908	0.0027							0.6906	0.0016
BAB1_1126	gi|82699945	Single-stranded DNA-binding protein	0.6942	0.0396	0.7899	0.0425								
BAB1_1129	gi|82699948	histone-like DNA-binding protein			0.409	0.0251	0.2368	0.0412	0.1671	0.025				
BAB1_1138	gi|82699956	chaperonin clpA/B			1.2688	0.0174							1.1977	0.0244
BAB1_1149	gi|82699966	dihydrolipoamide dehydrogenase	1.9972	0.0242	1.6034	0.0334	1.9563	0.0036	1.5518	0.0059	2.1108	0.0162	1.7003	0.0157
BAB1_1150	gi|82699967	branched-chain alpha-keto acid dehydrogenase subunit E2	1.5047	0.028	1.3913	0.0195	1.3685	0.0202	1.2414	0.0005	1.4244	0.0285	1.3176	0.0028
BAB1_1151	gi|82699968	pyruvate dehydrogenase subunit beta	2.1627	0.0004	1.7773	0.0005	2.3262	0.0002	1.8883	0.0002	1.9718	0.0007	1.6248	0.0006
BAB1_1152	gi|82699969	dehydrogenase, E1 component:mitochondrial substrate carrier	2.0036	0.001	1.9273	0.001	1.9098	0.001	1.8178	0.001	1.5091	0.0023	1.4612	0.0001
BAB1_1155	gi|82699972	enolase	2.0594	0.001	1.3469	0.0129					1.385	0.0074		
BAB1_1170	gi|82616088	citrate synthase I	1.2992	0.0249	1.2445	0.0207	1.3675	0.0008			1.5273	0.0026	1.4701	0.0032
BAB1_1181	gi|82699998	ribosome recycling factor	0.8251	0.012							0.7572	0.035		
BAB1_1184	gi|82700001	30S ribosomal protein S2	0.7401	0.0161	0.8666	0.0146					0.6072	0.0047	0.7119	0.0019
BAB1_1186	gi|82700002	ATP/GTP-binding motif-containing protein	0.6038	0.0052	0.7669	0.0423								
BAB1_1205	gi|62290094	hypothetical protein BAB1_1205							0.8225	0.0107	0.617	0.0114	0.589	0.0132
BAB1_1216	gi|82700028	TRAP transporter solute receptor, TAXI family					0.3989	0.0029	0.5305	0.0038	0.463	0.0098	0.6213	0.0168
BAB1_1221	gi|82700033	isocitrate dehydrogenase					1.426	0.0007	1.1889	0.0183				
BAB1_1223	gi|82700035	alanyl-tRNA synthetase					1.3054	0.0103			1.3559	0.0448		
BAB1_1251	gi|82700063	30S ribosomal protein S19	0.7766	0.0279	0.8075	0.0426								
BAB1_1252	gi|82700064	50S ribosomal protein L2	0.6043	0.0075	0.6385	0.0042								
BAB1_1254	gi|82616167	23S ribosomal protein L4	0.5688	0.0175	0.5201	0.0003								
BAB1_1263	gi|82700075	DNA-directed RNA polymerase subunit beta					1.2241	0.0104	1.2021	0.0039	1.295	0.0073	1.2944	0.0004
BAB1_1267	gi|91207342	50S ribosomal protein L1			0.7914	0.0183	0.8127	0.0456	0.7328	0.0052			0.8005	0.0162
BAB1_1269	gi|82700081	transcription antitermination protein NusG					0.6364	0.0006	0.7562	0.0011				
BAB1_1271	gi|82700083	elongation factor Tu					1.5003	0.0002	1.4303	0.0006	1.4575	0.0244	1.4094	0.001
BAB1_1283	gi|82700095	hypothetical protein BAB1_1283									0.634	0.0216	0.7299	0.0082
BAB1_1286	gi|82700098	glyoxalase/bleomycin resistance protein/dioxygenase			1.3544	0.0033	1.2502	0.0306	1.4183	0.0091			1.2606	0.0008
BAB1_1297	gi|82700106	hypothetical protein BAB1_1297	1.3179	0.0439	1.3793	0.0362								
BAB1_1327	gi|82700133	cobalamin synthesis protein P47K							1.2232	0.0181			1.246	0.0003
BAB1_1334	gi|82700139	3-hydroxyisobutyrate dehydrogenase			0.7527	0.0264			0.6593	0.039				
BAB1_1351	gi|82700154	sulfate-/thiosulfate-binding protein	0.4968	0.05	0.5137	0.002	0.4563	0.0194	0.4815	0.0009	0.2952	0.0088	0.296	0.0004
BAB1_1355	gi|82616259	hypothetical protein BAB1_1355					1.5569	0.0277	2.9565	0.0003				
BAB1_1356	gi|82700159	RbsD or FucU transport					0.7812	0.0038			0.773	0.0223		
BAB1_1392	gi|82700190	hypothetical protein BAB1_1392					0.5217	0.013	0.5189	0.0035			0.6641	0.0187
BAB1_1397	gi|82700195	aminotransferase, class I and II	0.7518	0.0365	0.7357	0.0038			0.7793	0.0173	0.7047	0.0397	0.6872	0.0023
BAB1_1413	gi|82700211	serine protease family protein									1.3192	0.0067	1.2865	0.0116
BAB1_1419	gi|82700217	hypothetical protein BAB1_1419			0.6569	0.0026	0.3901	0.0001	0.2704	0.0001			0.4584	0.0004
BAB1_1423	gi|82700221	hypothetical protein BAB1_1423			0.7325	0.0169							0.6595	0.0304
BAB1_1443	gi|82700241	UDP-3-O-[3-hydroxymyristoyl] N-acetylglucosamine deacetylase			1.2417	0.0278					1.3029	0.0227		
BAB1_1458	gi|88909188	S-adenosyl-L-methionine-dependent methyltransferase	1.6122	0.0016	1.5898	0.0004	1.3285	0.0128	1.2946	0.005	1.5748	0.0421	1.5584	0.0362
BAB1_1469	gi|82616368	ArsR family transcriptional regulator					0.5951	0.0116	0.6196	0.0025				
BAB1_1488	gi|82700282	choloylglycine hydrolase	1.188	0.0106							1.3365	0.0016	1.2749	0.0017
BAB1_1559	gi|82700351	Rieske iron-sulphur domain-containing protein	0.383	0.0317	0.4797	0.0476	0.349	0.0253	0.4325	0.0283	0.2953	0.0301	0.371	0.0342
BAB1_1583	gi|82616474	HMG-CoA lyase-like:Alpha-isopropylmalate/homocitrate synthase			0.7314	0.0435			0.6751	0.0043				
BAB1_1617	gi|82616506	Gfo/Idh/MocA family oxidoreductase					1.7183	0.0046	1.5211	0.0229				
BAB1_1644	gi|62290504	hypothetical protein BAB1_1644	0.6571	0.0386	0.6154	0.0103					0.6504	0.0037	0.4115	0.0383
BAB1_1647	gi|82700424	short chain dehydrogenase					0.7266	0.0384			0.7766	0.0306		
BAB1_1648	gi|82700425	periplasmic binding protein/LacI transcriptional regulator	0.5867	0.0011	0.5457	0.001	0.4888	0.0031	0.4517	0.0001	0.6335	0.0187	0.5931	0.001
BAB1_1671	gi|82700448	two-component response regulator	0.7793	0.0025	0.7636	0.0008					0.7302	0.0116	0.7189	0.0101
BAB1_1681	gi|82616559	tonB	5.5899	0.01	7.7212	0.01					8.9448	0.01	9.5222	0.01
BAB1_1697	gi|82700473	D-3-phosphoglycerate dehydrogenase	1.6456	0.0331	1.3984	0.0397					1.5512	0.0249	1.3204	0.0195
BAB1_1703	gi|82700479	peptidase M41	1.2959	0.0151							1.3936	0.0031		

BAB1_1709	gi|82700485	translocation protein TolB	0.8331	0.0318			0.5344	0.0006	0.6147	0.0001	0.6065	0.0026	0.7128	0.0004
BAB1_1719	gi|82700495	thiamine-phosphate pyrophosphorylase	1.6106	0.0007	1.2789	0.0362								
BAB1_1723	gi|82700499	inositol phosphatase/fructose-1,6-bisphosphatase									0.792	0.0059	0.7417	0.041
BAB1_1738	gi|82700514	hypothetical protein BAB1_1738	1.2774	0.0415	1.6026	0.0108							1.3964	0.0272
BAB1_1740	gi|82700516	transketolase					1.2298	0.0398	1.2203	0.0108			1.288	0.0234
BAB1_1742	gi|82698932	Phosphoglycerate kinase			1.4307	0.0014			1.4635	0.0004				
BAB1_1761	gi|82700535	pyruvate kinase					1.5337	0.0009	1.5879	0.0037	1.4168	0.0316	1.4911	0.0119
BAB1_1792	gi|82700565	Leu/Ile/Val-binding family protein	0.2519	0.0045	0.2408	0.0112	0.1702	0.0017	0.1611	0.0044	0.4732	0.0094	0.4449	0.0308
BAB1_1800	gi|82700572	proline racemase, putative					0.5172	0.05	0.444	0.0454				
BAB1_1808	gi|82700580	F0F1 ATP synthase subunit gamma									1.5348	0.0147	1.5555	0.0228
BAB1_1809	gi|82700581	F0F1 ATP synthase subunit alpha					1.3905	0.0035	1.5078	0.0057			1.2744	0.0061
BAB1_1810	gi|82700582	F0F1 ATP synthase subunit delta					0.4768	0.0207	0.5313	0.0069			0.7675	0.0135
BAB1_1827	gi|82700597	NAD-glutamate dehydrogenase	0.7096	0.0008	0.6963	0.001	0.6941	0.0005	0.6685	0.0004	0.8137	0.0036	0.8021	0.008
BAB1_1845	gi|82616716	carboxyl-terminal protease			1.4928	0.0002	1.2267	0.0202	1.3653	0.0126				
BAB1_1868	gi|82700638	chaperonin ClpA/B									0.7212	0.015	0.6938	0.001
BAB1_1872	gi|82616743	peptide chain release factor 1			1.4335	0.0001	1.3547	0.0067	1.6533	0.0006			1.2946	0.0065
BAB1_1875	gi|82700645	3-demethylubiquinone-9 3-methyltransferase	0.6804	0.0014	0.7594	0.044	0.5134	0.0008	0.5602	0.0007	0.5618	0.0117	0.6207	0.0008
BAB1_1890	gi|82700658	YciI-like protein							0.7153	0.0334	0.6924	0.0179	0.6827	0.0237
BAB1_1904	gi|82700672	GCN5-related N-acetyltransferase					0.5926	0.0116	0.6374	0.0007			0.7254	0.0406
BAB1_1922	gi|82700688	dihydrolipoamide acetyltransferase	1.6115	0.0001	1.3788	0.0037	2.1834	0.0002	1.8479	0.0003	1.4219	0.0013	1.2219	0.0022
BAB1_1923	gi|82700689	alpha-ketoglutarate decarboxylase									1.2391	0.0334	1.265	0.001
BAB1_1926	gi|82616792	Succinyl-CoA synthetase subunit beta					1.564	0.001	1.5872	0.001				
BAB1_1944	gi|82700707	PpiC-type peptidyl-prolyl cis-trans isomerase			1.2893	0.0358	1.2776	0.0168	1.3463	0.01				
BAB1_1968	gi|82700728	glucose/ribitol dehydrogenase	0.4768	0.0174	0.5162	0.0028	0.348	0.0055	0.3705	0.0005	0.4466	0.0119	0.485	0.0011
BAB1_1970	gi|82700729	3-hydroxybutyryl-CoA dehydrogenase	1.2181	0.0328	1.202	0.0026	1.2074	0.0435					1.2693	0.0172
BAB1_1971	gi|82700730	antifreeze protein, type I:electron transfer flavoprotein, alpha subunit	1.3432	0.016	1.5035	0.0039	1.4836	0.0001	1.6448	0.0051	1.3539	0.0191	1.5205	0.0082
BAB1_1972	gi|82700731	electron transfer flavoprotein subunit beta			1.6426	0.0007							1.4822	0.0011
BAB1_2059	gi|82700813	ParB-like nuclease:ParB-like partition protein					0.8105	0.0046	0.8115	0.0039	0.7819	0.0447	0.7925	0.0381
BAB1_2107	gi|82700858	thioredoxin domain-containing protein	0.415	0.0013	0.4558	0.0016	0.4791	0.0007	0.5203	0.0006	0.3753	0.0004	0.4144	0.0009
BAB1_2124	gi|82700871	50S ribosomal protein L20	0.5398	0.0105	0.632	0.0149								
BAB1_2130	gi|82700877	cytochrome c heme-binding domain-containing protein	0.4892	0.0299	0.4692	0.0029							0.5819	0.0471
BAB1_2133	gi|82700880	hypothetical protein BAB1_2133					0.6296	0.0466	0.5937	0.0334				
BAB1_2150	gi|82700896	DNA starvation/stationary phase protection protein Dps	0.5403	0.0012							0.4854	0.0011	0.7548	0.0107
BAB1_2163	gi|82700909	transcription elongation factor NusA			1.2381	0.003			1.2684	0.0181				
BAB1_2175	gi|82617022	Ferric-uptake regulator	3.1651	0.01	4.288	0.01					3.5608	0.01	4.8458	0.01
BAB1_2176	gi|82700922	NLPA lipoprotein			0.8005	0.0352					0.6248	0.0381		
BAB2_0015	gi|82939245	Isochorismate synthase	4.7656	0.01	6.8178	0.01					4.5681	0.01	6.5649	0.01
BAB2_0023	gi|82939253	putative branched chain amino acid ABC transporter, periplasmic amino acid-binding protein			0.8054	0.0287			0.8024	0.0286				
BAB2_0113	gi|83269064	solute-binding family 1 protein	1.2213	0.0074							1.2762	0.0232		
BAB2_0177	gi|83269116	aldo/keto reductase					0.6896	0.001	0.6253	0.001				
BAB2_0195	gi|83269133	hypothetical protein BAB2_0195	0.5503	0.0131			0.7146	0.0124						
BAB2_0198	gi|82939410	hypothetical protein BAB2_0198					1.2966	0.0048	1.2647	0.0063				
BAB2_0261	gi|83269185	RecA DNA recombination protein:alanine dehydrogenase/PNT			1.2538	0.0305							1.2746	0.036
BAB2_0275	gi|83269197	YaeC family lipoprotein	0.6928	0.0097	0.7046	0.0001	0.6075	0.0044	0.6068	0.001	0.4608	0.0012	0.461	0.001
BAB2_0282	gi|83269204	Leu/Ile/Val-binding family protein	0.3681	0.0034	0.3208	0.0045	0.3311	0.0054	0.2832	0.003			0.5022	0.0234
BAB2_0294	gi|83269216	dihydroxy-acid dehydratase	0.6255	0.0279	0.6223	0.0477								
BAB2_0295	gi|83269217	2-keto-3-deoxy-galactonokinase									0.5447	0.0032	0.6032	0.0183
BAB2_0327	gi|83269246	aldehyde dehydrogenase	0.509	0.0002	0.4623	0.0039	0.4443	0.0002	0.4044	0.0019				
BAB2_0338	gi|83269254	ornithine cyclodeaminase			0.5711	0.0119			0.5603	0.0239				
BAB2_0346	gi|83269262	3-isopropylmalate dehydrogenase			1.2914	0.0463					1.6265	0.0417	1.4601	0.0385
BAB2_0358	gi|82939548	peptidyl-dipeptidase DCP			0.7101	0.0061	0.6306	0.0001	0.5323	0.0001	0.5843	0.0001	0.4986	0.001
BAB2_0364	gi|83269280	fructose-1,6-bisphosphatase			1.8809	0.0462							2.3821	0.0312
BAB2_0365	gi|83269281	fructose-1,6-bisphosphate aldolase	1.7151	0.0166	1.813	0.0124	2.0037	0.0117	2.0972	0.0108	1.7059	0.0235	1.8084	0.021
BAB2_0459	gi|83269362	6-phosphogluconolactonase	0.6583	0.0214							0.5931	0.0286		
BAB2_0460	gi|83269363	glucose-6-phosphate 1-dehydrogenase	0.7572	0.0003	0.7225	0.0082	0.7989	0.0147	0.7533	0.0054	0.6	0.0016	0.5699	0.0001
BAB2_0491	gi|83269394	extracellular solute-binding protein	1.757	0.0206	1.7232	0.0082							1.3722	0.0322

BAB2_0513	gi|83269412	glycine cleavage system aminomethyltransferase T			0.6442	0.0356	0.6765	0.0252	0.625	0.0134	0.5011	0.0176	0.463	0.0044
BAB2_0518	gi|83269417	bifunctional proline dehydrogenase/pyrroline-5-carboxylate dehydrogenase	0.7368	0.0276	0.7293	0.0452								
BAB2_0531	gi|83269430	Alkyl hydroperoxide reductase/ Thiol specific antioxidant/ Mal allergen	1.6028	0.003	1.453	0.0008	2.8073	0.0002	2.5263	0.0021	1.636	0.0164	1.4883	0.0008
BAB2_0532	gi|82939705	alkylhydroperoxidase, AhpD family					1.563	0.0193	1.3917	0.05				
BAB2_0535	gi|83269434	copper/Zinc superoxide dismutase			2.3811	0.0003							1.8662	0.0018
BAB2_0539	gi|83269436	solute-binding family 1 protein	2.9779	0.0239	3.4135	0.0003	3.1378	0.0131	3.5434	0.0004	3.2462	0.0419	3.6957	0.0004
BAB2_0545	gi|83269442	riboflavin synthase subunit beta					0.583	0.0045	0.7417	0.0513				
BAB2_0547	gi|82939718	Probable sugar-binding periplasmic protein precursor	0.3804	0.0001	0.3435	0.001	0.3118	0.001	0.2798	0.001	0.457	0.0004	0.4131	0.0001
BAB2_0558	gi|83269453	glutamate-binding protein			1.7854	0.0004							1.7467	0.0001
BAB2_0564	gi|82939733	iron compound ABC transporter, periplasmic iron compound-binding protein	2.1714	0.0021	2.4269	0.0025	2.0984	0.0005	2.3466	0.0008	2.152	0.0009	2.4347	0.0011
BAB2_0585	gi|83269478	solute-binding family 1 protein	0.6375	0.0153	0.6495	0.0001					0.6525	0.0256	0.6619	0.001
BAB2_0620	gi|83269506	hypothetical protein BAB2_0620			0.7769	0.0019							0.6023	0.026
BAB2_0665	gi|83269549	hypothetical protein BAB2_0665					0.323	0.0009	0.392	0.0059	0.5704	0.0183	0.687	0.0424
BAB2_0675	gi|83269559	Bacterioferritin	0.3436	0.0012	0.3745	0.0008	0.5295	0.0053	0.5802	0.0019	0.3466	0.0083	0.3774	0.0014
BAB2_0700	gi|83269583	solute-binding family 5 protein			1.3806	0.003							1.2743	0.0168
BAB2_0740	gi|83269621	hypothetical protein BAB2_0740	0.8271	0.0274			0.7184	0.0058						
BAB2_0759	gi|83269638	hypothetical protein BAB2_0759					0.4443	0.0026	0.4532	0.0059	0.6256	0.0111	0.6477	0.019
BAB2_0840	gi|82939977	Hypothetical protein, conserved	2.8728	0.0495	3.7423	0.0125							2.6159	0.0086
BAB2_0848	gi|83269711	catalase			0.8269	0.0309	0.5198	0.0239	0.5487	0.0001			0.6309	0.0089
BAB2_0851	gi|83269714	inosine 5'-monophosphate dehydrogenase					0.7422	0.0058	0.746	0.0261	0.6217	0.0092	0.6256	0.0128
BAB2_0880	gi|83269741	hypothetical protein BAB2_0880					0.5589	0.0359	0.4574	0.0011				
BAB2_0904	gi|83269759	nitrate reductase alpha subunit	0.7038	0.0467	0.6435	0.0145			0.7753	0.0438	0.499	0.0463	0.4588	0.0378
BAB2_0919	gi|83269772	sulfonate/nitrate ABC transporter periplasmic-binding protein			2.2931	0.0243					3.0307	0.0408	3.1244	0.0182
BAB2_0938	gi|83269790	periplasmic binding protein/LacI transcriptional regulator	0.3734	0.0002	0.4073	0.001	0.3521	0.0001	0.3878	0.001	0.3964	0.0006	0.431	0.0001
BAB2_0974	gi|83269821	solute-binding family 5 protein	0.8187	0.04			0.6317	0.0354	0.7007	0.0106	0.731	0.0272		
BAB2_0975	gi|83269822	3-ketoacyl-(acyl-carrier-protein) reductase	0.5451	0.0082	0.7392	0.0314	0.5717	0.0033			0.4118	0.0027	0.5302	0.0007
BAB2_1009	gi|83269853	methylglyoxal synthase					0.704	0.0007	0.6841	0.0289				
BAB2_1033	gi|83269876	GntR family regulatory protein							0.6285	0.017			0.7832	0.0415
BAB2_1146	gi|83269977	solute-binding family 1 protein					0.4636	0.0411			0.54	0.002	0.7026	0.0227
BAB2_1153	gi|83269984	Leu/Ile/Val-binding family protein					0.5835	0.0046	0.7406	0.0091	0.6537	0.007		
BAB2_1163	gi|83269990	NifH/FrxC family ATPase					1.3404	0.0062			1.4286	0.0247		

Supplementary Table S2: Amino acid transport and metabolism (E)[a]

NCBI GI	Gene ID	Gene	Protein description	Loc [b]	Signal peptide	Mean ratio(I/C)	SD	Ratio
GI:82615038	BAB1_0005		D-isomer specific 2-hydroxyacid dehydrogenase NAD-binding	C	NO	0.79	0.02	↓
GI:82698997	BAB1_0071		argininosuccinate synthase	C	NO	0.69	0.10	↓
GI:82699018	BAB1_0096	ilvD	dihydroxy-acid dehydratase	C	NO	0.52	0.09	↓
GI:82699040	BAB1_0118		Peptidase M20/M25/M40	C	NO	1.39	0.16	↑
GI:82615286	BAB1_0285	hisD	Histidinol dehydrogenase	C	NO	1.26	0.06	↑
GI:82699525	BAB1_0657		histidinol-phosphate aminotransferase	C	NO	1.67	0.25	↑
GI:82699578	BAB1_0710		leucyl aminopeptidase	C	NO	0.75	0.06	↓
GI:82615747	BAB1_0787	glyA	Serine hydroxymethyltransferase	C	NO	1.44	0.01	↑
GI:82699672	BAB1_0813		O-acetylhomoserine aminocarboxypropyltransferase	C	NO	0.69	0.10	↓
GI:82699902	BAB1_1073		cysteine synthase A	C	NO	0.76	0.06	↓
GI:82700098	BAB1_1286	gloA	glyoxalase/ dioxygenase	C	NO	1.32	0.08	↑
GI:82700195	BAB1_1397		aminotransferase, class I and II	C	NO	0.73	0.04	↓
GI:82616474	BAB1_1583	leuA	2-isopropylmalate synthase	C	NO	0.70	0.04	↓
GI:82700473	BAB1_1697		D-3-phosphoglycerate dehydrogenase	C	NO	1.48	0.15	↑
GI:82700565	BAB1_1792		Leu/Ile/Val-binding family	P	NO	0.29	0.14	↓
GI:82700572	BAB1_1800		proline racemase	C	NO	0.48	0.05	↓
GI:82700597	BAB1_1827		Bacterial NAD-glutamate dehydrogenase	CM	NO	0.73	0.06	↓
GI:82700728	BAB1_1968		glucose/ribitol dehydrogenase	C	NO	0.44	0.07	↓
GI:82939253	BAB2_0023		Leu/Ile/Val-binding family protein	P	NO	0.80	0.01	↓
GI:83269204	BAB2_0282		Leu/Ile/Val-binding family protein	U	YES	0.36	0.08	↓
GI:83269216	BAB2_0294		dihydroxy-acid dehydratase	C	NO	0.62	0.01	↓
GI:83269254	BAB2_0338		ornithine cyclodeaminase	C	NO	0.57	0.01	↓
GI:83269262	BAB2_0346		3-isopropylmalate dehydrogenase	C	NO	1.46	0.17	↑
GI:82939548	BAB2_0358	dcp	peptidyl-dipeptidase DCP	C	NO	0.59	0.08	↓
GI:83269412	BAB2_0513	gcvT	Glycine cleavage system protein	C	NO	0.58	0.09	↓
GI:83269453	BAB2_0558		glutamate-binding protein	P	NO	1.77	0.03	↑
GI:83269583	BAB2_0700		ABC-type oligopeptide transport system, periplasmic component	P	YES	1.33	0.08	↑
GI:83269821	BAB2_0974		ABC-type dipeptide transport system, periplasmic component	P	YES	0.72	0.08	↓
GI:83269984	BAB2_1153		Leu/Ile/Val-binding family protein	P	NO	0.66	0.08	↓

a) Abbreviations of assigned functional categories (http://www.ncbi.nih.gov/COG/).

b) Abbreviations of cellular location. Protein cellular location was annotated by PSORTB v. 3.0 (http://www.psort.org/). C, cytoplasmic; P, periplasmic; U, unknown; CM, cytoplasmic membrane.

Mean ratio (I/C), indicates the mean values of all I/C ratios with $P<0.05$.

SD, indicates standard deviation of the mean.

Supplementary Table S3: Hypothetical proteins, function unknown and general function predicted only (S, R)[(a)].

NCBI GI	Gene ID	Protein predicted	Loc[b)]	Signal peptide	Mean ratio(I/C)	SD	Ratio
**Hypothetical proteins**							
GI:82699001	BAB1_0075	-	P	YES	0.39	0.09	↓
GI:82699635	BAB1_0776	-	U	YES	1.37	0.13	↑
GI:82700002	BAB1_1186	ATP/GTP binding motif containing protein	U	YES	0.69	0.12	↓
GI:62290094	BAB1_1205	-	U	NO	0.68	0.13	↓
GI:82616259	BAB1_1355	-	U	YES	2.26	0.99	↑
GI:62290504	BAB1_1644	-	U	NO	0.58	0.12	↓
GI:82700514	BAB1_1738	-	U	NO	1.43	0.16	↑
GI:83269133	BAB2_0195	-	U	NO	0.63	0.12	↓
GI:83269506	BAB2_0620	-	C	NO	0.69	0.12	↓
GI:83269741	BAB2_0880	-	U	NO	0.51	0.07	↓
**Function unknown (S)**							
GI:82699830	BAB1_0993	-	U	NO	2.59	0.94	↑
GI:82699861	BAB1_1029	-	U	YES	0.71	0.07	↓
GI:82700095	BAB1_1283	-	U	YES	0.68	0.07	↓
GI:82700106	BAB1_1297	-	C	NO	1.35	0.04	↑
GI:82700217	BAB1_1419	-	U	NO	0.44	0.16	↓
GI:82700221	BAB1_1423	-	C	NO	0.70	0.05	↓
GI:82700658	BAB1_1890	YciI-like protein	U	NO	0.70	0.02	↓
GI:82700880	BAB1_2133	-	U	NO	0.61	0.03	↓
GI:83269638	BAB2_0759	-	C	NO	0.54	0.11	↓
**General function predicted only (R)**							
GI:82699116	BAB1_0204	Zinc containing alcohol dehydrogenase	C	NO	0.47	0.15	↓
GI:82699260	BAB1_0368	Invasion protein B	U	YES	0.49	0.15	↓
GI:82700028	BAB1_1216	Trap transporter solute receptor, Taxi family	U	YES	0.50	0.10	↓
GI:82700133	BAB1_1327	Cobalamin synthesis protein	C	NO	1.23	0.02	↑
GI:82700190	BAB1_1392	-	C	NO	0.57	0.08	↓
GI:82616506	BAB1_1617	Gfo/Idh/MocA family oxidoreductase	U	NO	1.62	0.14	↑
GI:82700672	BAB1_1904	GCN5-related N-acetyltransferase	C	NO	0.65	0.07	↓
GI:83269116	BAB2_0177	aldo/keto reductase	C	NO	0.66	0.05	↓
GI:83269549	BAB2_0665	-	U	YES	0.49	0.17	↓
GI:83269621	BAB2_0740	-	U	NO	0.77	0.06	↓

[a)] Abbreviations of assigned functional categories (http://www.ncbi.nih.gov/COG/).
[b)] Abbreviations of cellular location. Protein cellular location was annotated by PSORTB v. 3.0 (http://www.psort.org/). C, cytoplasmic; P, periplasmic; U, unknown; CM, cytoplasmic membrane.
Mean ratio (I/C), indicates the mean values of all I/C ratios with *P*<0.05.
SD, indicates standard deviation of the mean.

Supplementary Table S4: Others Cogs

NCBI GI	Gene ID	Gene	Protein description	Loc. (b)	Signal peptide	Mean ratio(I/C)	SD	Ratio
**Cell wall/membrane/envelope biogenesis (M)**								
GI:82699528	BAB1_0660	Omp2b	Omp2b porin	OM	NO	1.39	0.21	↑
GI:82700241	BAB1_1443	lpxC	UDP-3-O-[3-hydroxymyristoyl] N-acetylglucosamine deacetylase	C	NO	1.27	0.04	↑
GI:88909188	BAB1_1458	mraW	S-adenosyl-L-methionine-dependent methyltransferase	C	NO	1.49	0.14	↑
GI:82700282	BAB1_1488	cgh	choloylglycine hydrolase	U	NO	1.27	0.07	↑
GI:82616716	BAB1_1845	ctpA	carboxyl-terminal protease	CM	NO	1.36	0.13	↑
**Cell cycle control, cell division, chromosome partitioning (D)**								
GI:83269990	BAB2_1163		NifH/FrxC family ATPase	C	NO	1.38	0.06	↑
**Coenzyme transport and metabolism (H)**								
GI:82699123	BAB1_0215	thiE	thiamine-phosphate pyrophosphorylase	C	NO	0.56	0.05	↓
GI:82699639	BAB1_0780	hemB	delta-aminolevulinic acid dehydratase	C	NO	0.73	0.07	↓
GI:82616368	BAB1_1469		ArsR family transcriptional regulator	C	NO	0.61	0.02	↓
GI:82700495	BAB1_1719		thiamine-phosphate pyrophosphorylase	C	NO	1.44	0.23	↑
GI:82700645	BAB1_1875	ubiG	3-demethylubiquinone-9 3-methyltransferase	C	NO	0.62	0.09	↓
GI:83269442	BAB2_0545	ribH-2	riboflavin synthase subunit beta	C	NO	0.66	0.11	↓
**Intracellular trafficking, secretion, and vesicular transport (U)**								
GI:82700485	BAB1_1709	tolB	translocation protein TolB	P	NO	0.66	0.12	↓
**Lipid transport and metabolism (I)**								
GI:82699370	BAB1_0484	acpP	acyl carrier protein	C	NO	1.80	0.43	↑
GI:82699727	BAB1_0873		3-oxoacyl-(acyl carrier protein) synthase II	U	NO	0.67	0.05	↓
GI:83269822	BAB2_0975		3-ketoacyl-(acyl-carrier-protein) reductase	C	NO	0.56	0.12	↓
GI:82699928	BAB1_1109		acyl-CoA dehydrogenase	C	NO	0.59	0.10	↓
GI:82700139	BAB1_1334		3-hydroxyisobutyrate dehydrogenase	C	NO	0.71	0.07	↓
GI:82700424	BAB1_1647		short chain dehydrogenase	C	NO	0.75	0.04	↓
GI:82700729	BAB1_1970		3-hydroxybutyryl-CoA dehydrogenase	C	NO	1.22	0.03	↑
**Nucleotide transport and metabolism (F)**								
GI:82699556	BAB1_0688	pyrC-1	dihydroorotase	C	NO	1.40	0.12	↑
GI:82699711	BAB1_0857	purL	phosphoribosylformylglycinamidine synthase II	C	NO	1.35	0.11	↑
GI:82699722	BAB1_0868	purB	adenylosuccinate lyase	C	NO	1.32	0.04	↑
GI:83269714	BAB2_0851		inosine 5'-monophosphate dehydrogenase	C	NO	0.68	0.07	↓
**Replication, recombination and repair (L)**								
GI:82698934	BAB1_0002	dnaN	DNA polymerase III subunit beta	C	NO	1.31	0.09	↑
GI:82699658	BAB1_0799	ihfA	integration host factor subunit alpha	C	NO	1.42	0.12	↑
GI:82699847	BAB1_1015	tatD	TatD-related deoxyribonuclease	C	NO	1.52	0.15	↑
GI:82699945	BAB1_1126	ssb	Single-stranded DNA-binding protein	C	NO	0.74	0.07	↓
GI:82699948	BAB1_1129		histone-like DNA-binding protein	C	NO	0.27	0.12	↓
**Signal transduction mechanisms (T)**								
GI:82699504	BAB1_0636		transcriptional regulatory protein, C terminal	C	NO	0.72	0.11	↓
GI:82699897	BAB1_1068	uspA	universal stress protein	C	NO	1.61	0.19	↑
GI:82699956	BAB1_1138	clpA/B	chaperonin clpA/B	C	NO	1.23	0.05	↑
GI:82700448	BAB1_1671		two-component response regulator receiver	C	NO	0.75	0.03	↓
**Secondary metabolites biosynthesis, transport and catabolism (Q)**								
GI:82699370	BAB1_0484	acpP	acyl carrier protein	C	NO	1.80	0.43	↑

GI:82699727	BAB1_0873		3-oxoacyl-(acyl carrier protein) synthase II	U	NO	0.67	0.05	↓
GI:83269822	BAB2_0975		3-ketoacyl-(acyl-carrier-protein) reductase	C	NO	0.56	0.12	↓
GI:82700424	BAB1_1647		short chain dehydrogenase	C	NO	0.75	0.04	↓
**Transcription (K)**								
GI:82699504	BAB1_0636		transcriptional regulatory protein, C terminal	C	NO	0.72	0.11	↓
GI:91208373	BAB1_0788	nrdR	Transcriptional repressor nrdR	C	NO	1.24	0.03	↑
GI:82700075	BAB1_1263	rpoC	DNA-directed RNA polymerase subunit beta	C	NO	1.25	0.05	↑
GI:82700081	BAB1_1269	nusG	transcription antitermination protein NusG	C	NO	0.70	0.08	↓
GI:82700195	BAB1_1397		aminotransferase, class I and II	C	NO	0.73	0.04	↓
GI:82700813	BAB1_2059		ParB-like nuclease:ParB-like partition protein	C	NO	0.80	0.01	↓
GI:82700909	BAB1_2163	nusA	transcription elongation factor NusA	C	NO	1.25	0.02	↑
GI:83269876	BAB2_1033		GntR family regulatory protein	C	NO	0.71	0.11	↓

[a] Abbreviations of assigned functional categories (http://www.ncbi.nih.gov/COG/).

[b] Abbreviations of cellular location. Protein cellular location was annotated by PSORTB v. 3.0 (http://www.psort.org/). C, cytoplasmic; P, periplasmic; U, unknown; CM, cytoplasmic membrane; OM, outer membrane.

Mean ratio (I/C), indicates the mean values of all I/C ratios with $P<0.05$.

SD, indicates standard deviation of the mean.

Supplementary Table S5

	Protein name	Putative ICE motif a)	Position b)
**Iron uptake**			
BAB1_1681	TonB	**nf**	
BAB2_0015	EntC	**nf**	
BAB2_0113		**nf**	
BAB2_0539	FbpA	**GAGTGGCAATTGCGCAAAACTGG**	-45*
BAB2_0564	FatB	**GTTCGGCTCTTTCGCGAGCTGT**	-193
BAB2_0840		**nf**	
**Iron storage**			
BAB1_2150	Dps	**nf**	
BAB2_0675	Bfr	**CGGTTAGAATTATTCTAAATA**	-308*
**Transcription factors**			
BAB1_2175	Irr	**nf**	
BAB1_0799	Ihfα	**TTGCTGGAAATGCTCTAGTCG**	-22*
**Iron utilization pathways**			
Iron sulfur proteins			
BAB1_0090	AcnA	**TTTGCGTGGGGTTTAAA**	-318
BAB1_0096	IlvD	**AAGTTAGAATAGTTCCTAAAA**	-10
BAB1_0139	NifU	**nf**	
BAB1_0825	NuoD	**nf**	
BAB1_0826	NuoE	**nf**	
BAB1_0827	NuoF	**nf**	
BAB1_0828	NuoG	**nf**	
BAB1_0855	Grx	**nf**	
BAB2_0023	Branched chain amino acid ABC transporter	**GTTTGACCAGTTAAACCATCTTC**	-48
BAB2_0294	IlvD	**nf**	
BAB2_0848	Cat	**TTATTGGAATAGGTCTAATTG**	-102
BAB2_0904	NarG	**nf**	
BAB2_1163	NifH	**nf**	
Heme containing proteins			
BAB1_0036	CitC	**TTTATCGACAGGGAAAA**	-190
BAB1_0389	CcoP	**nf**	
Iron containing proteins			
BAB1_1559	PetA	**GAGTTAGAGCGGCTCCTGTTG**	-122
Synthesis of Fe-S			
BAB1_0139	nifU	**nf**	
BAB1_0948	SufB	**AGTTTAGAGAGGTTTTAAAGA**	-99
**Other or uncertain functional roles**			
BAB1_0075	HP	**GTTCCGTGATTATACCAGGCTGT**	-51
BAB1_0104	sulfate-thiosulfate binding protein	**TCATTATAGCGGTTCCAGTCG**	-166
BAB1_0211	ALDH	**CCCTTGCAAATTTGCCACACTTT**	-59
BAB1_0238	ABC- transporter	**TATACGAACAGCGTAAA**	-126

BAB1_0591	SodA	**nf**	
BAB1_0660	Omp2b	**TATATGGCGCGCTTAAA**	-425
BAB1_0868	purB	**TCGTTGGAACCGCTCTAAGTG**	-82*
BAB1_0873	FabF	**ATTTTGGAAATGTTTTCAAAA**	-171*
BAB1_0930	RibEG	**TTGAACGAGGCCATAAA**	-473
BAB1_1068	UspA	**GTTTTGCCGCCTTTCCAAAGCTT**	-73
BAB1_1129	histone-like DNA binding protein	**AGCTTGATATTGTACTAAATG**	-174*
BAB1_1184	rpsB	**CAGTTTTTGCCGTTCCAGCCA**	-263
BAB1_1413	degP	**TGGTTAGAGCGGTTCCAGCGA**	-71
BAB1_1423	HP	**AATTTGACATCATTGTCAAAT**	-44*
BAB1_1648	ABC transporter	**TACTTAGAGCGGTTCCAGCGA**	-135
BAB1_1723	FBP	**ATTTTGGAACGGGTTCCAAGG**	-96*
BAB1_1875	UbiG	**TTTTAGCTATGTGACCACAAGTT**	-123
BAB1_1904	GCN5-related N-acetyltransferase	**TCCTTTGAACGGTTCTTAATT**	-10
BAB1_1970	FadB	**TTGCTGGAAATGCTCTAGTCG**	-63
BAB2_0195	HP	**ATATTAGAGCGGTTCCAACGA**	-149
BAB2_0338	Ocd	**ATATTAGAAGGAATCCAATTT**	-1*
BAB2_0346	IMDH	**TTTAAGCTGTAGTTAAA**	-30*
BAB2_0358	DCP	**nf**	
BAB2_0531	AhpC	**nf**	
BAB2_0535	SodC	**TAGTTAGAACAGTTCCAGAGA**	-16
BAB2_0880	HP	**TATTTATAGTGACTACAGATC**	-64
BAB2_0975	FabG	**GATCTAGAAACCCTCCCAATC**	-34*

[a)] Irr-binding motif occurrence on the intergenic regions of modulated *B. abortus* 2308 proteins was determined using the Find Individual Motif Occurrences (FIMO) algorithm software[47] using, nnnyTwkArnsryTCyArwyn[46], wdTTTrGAAynrTTCyAAAmw[46], AnnTTAGAAynrTTCTAAnnn[46], AnnTTrGAAynrTTCyAAAnn[46], TTTAN9-TAAA[26] and GTTTGGCAATTTACCAAACTTT[45] as a query sequences. *P*<0.0001.

[b)] Position relative to the start of translation.

* Predicted operon

nf: not found
